# Technology-Assisted Buprenorphine Treatment in Rural and Nonrural Settings: Two Randomized Clinical Trials

**DOI:** 10.1001/jamanetworkopen.2023.31910

**Published:** 2023-09-27

**Authors:** Stacey C. Sigmon, Kelly R. Peck, Sydney R. Batchelder, Gary J. Badger, Sarah H. Heil, Stephen T. Higgins

**Affiliations:** 1 Vermont Center on Behavior and Health, University of Vermont, Burlington; 2 Department of Psychiatry, University of Vermont, Burlington; 3 Department of Psychological Science, University of Vermont, Burlington; 4 Department of Medical Biostatistics, University of Vermont, Burlington

## Abstract

**Importance:**

Expansion of opioid use disorder treatment is needed, particularly in rural communities.

**Objective:**

To evaluate technology-assisted buprenorphine (TAB) efficacy (1) over a longer period than previously examined, (2) with the addition of overdose education, and (3) among individuals residing in rural communities.

**Design, Setting, and Participants:**

Two parallel, 24-week randomized clinical trials were conducted at the University of Vermont between February 1, 2018, and June 30, 2022. Participants were adults with untreated opioid use disorder from nonrural (trial 1) or rural (trial 2) communities. These trials are part of a programmatic effort to develop TAB protocols to improve treatment availability in underserved areas.

**Interventions:**

Within each trial, 50 participants were randomized to TAB or control conditions. Participants in the TAB group completed bimonthly visits to ingest medication and receive take-home doses via a computerized device. They received nightly calls via an interactive voice response (IVR) system, IVR-generated random call-backs, and iPad-delivered HIV, hepatitis C virus (HCV), and overdose education. Control participants received community resource guides and assistance with contacting resources. All participants received harm reduction supplies and completed monthly assessments.

**Main Outcomes and Measures:**

The primary outcome was biochemically verified illicit opioid abstinence across monthly assessments. Secondary outcomes included self-reported opioid use in both groups and abstinence at bimonthly and random call-back visits, treatment adherence, satisfaction, and changes in HIV, HCV, and overdose knowledge among TAB participants.

**Results:**

Fifty individuals (mean [SD] age, 40.6 [13.1] years; 28 [56.0%] male) participated in trial 1, and 50 (mean [SD] age, 40.3 [10.8] years; 30 [60.0%] male) participated in trial 2. Participants in the TAB group achieved significantly greater illicit opioid abstinence vs controls at all time points in both trial 1 (85.3% [128 of 150]; 95% CI, 70.7%-93.3%; vs 24.0% [36 of 150]; 95% CI, 13.6%-38.8%) and trial 2 (88.0% [132 of 150]; 95% CI, 72.1%-95.4%; vs 21.3% [32 of 150]; 95% CI, 11.4%-36.5%). High abstinence rates were also observed at TAB participants’ bimonthly dosing visits (83.0% [95% CI, 67.0%-92.0%] for trial 1 and 88.0% [95% CI, 71.0%-95.0%] for trial 2). Treatment adherence was favorable and similar between trials (with rates of approximately 99% for buprenorphine administration, 93% for daily IVR calls, and 92% for random call-backs), and 183 of 187 urine samples (97.9%) tested negative for illicit opioids at random call-backs. iPad-delivered education was associated with significant and sustained increases in HIV, HCV, and overdose knowledge.

**Conclusions and Relevance:**

In these randomized clinical trials of TAB treatment, demonstration of efficacy was extended to a longer duration than previously examined and to patients residing in rural communities.

**Trial Registration:**

ClinicalTrials.gov Identifier: NCT03420313

## Key Points

**Question:**

Does technology-assisted buprenorphine (TAB) treatment delivered over 24 weeks reduce illicit opioid use among individuals residing in nonrural (trial 1) and rural (trial 2) communities?

**Findings:**

In 2 randomized clinical trials that each included 50 participants with untreated opioid use disorder, participants in both nonrural and rural communities randomized to TAB achieved significantly higher rates of illicit opioid abstinence (85% and 88%, respectively) vs controls (21% and 24%, respectively).

**Meaning:**

These trials demonstrate the efficacy of TAB treatment in nonrural and rural patients.

## Teaser

These 2 randomized clinical trials investigate the efficacy of technology-assisted buprenorphine with longer treatment duration and with the addition of overdose education among individuals in rural and nonrural communities.

## Introduction

The US opioid epidemic remains a severe public health crisis exacerbated by the recent emergence of fentanyl and other potent synthetic analogues.^1,2,3,4,5^ Despite the efficacy of agonist therapy, only 22% of Americans with opioid use disorder (OUD) received treatment in the past year.^6^ Buprenorphine is highly effective and increasingly available in general medical settings yet remains significantly underused, especially in rural areas, where more than half of counties lack a single buprenorphine prescriber.^7,8,9,10,11,12,13,14,15^ Practitioners who prescribe buprenorphine also often treat far fewer patients than permitted in part because of concerns about potential medication nonadherence (eg, misuse, illicit diversion, and inadequate medication safekeeping).^13,16,17^ By the early 2000s, an alarming number of clinics had lengthy waiting lists for treatment, and individuals often remained on waiting lists for months or years, during which they were at risk of premature death.^18^

One effort to reduce drug use and related risks among patients on waiting lists was to offer interim methadone treatment, in which methadone clinics provided medication without formal counseling on a temporary basis.^19,20,21^ Building on the efficacy of interim methadone treatment,^22^ we sought to develop an interim buprenorphine protocol that could bridge treatment delays and geographic barriers without the regulatory and safety issues associated with methadone.^21,23^ We incorporated several mobile health (mHealth) components to support medication adherence and harm reduction.^24,25,26,27^ Technology-assisted buprenorphine (TAB) treatment consisted of 4 components: (1) bimonthly (ie, every 2 weeks) visits to provide a urine specimen, ingest medication, and receive the remaining doses for home administration via a portable computerized device; (2) nightly check-in calls via an interactive voice response (IVR) telephone system; (3) bimonthly IVR-generated random call-backs; and (4) iPad-administered HIV and hepatitis C virus (HCV) education. In a 12-week randomized clinical trial (RCT) among waitlisted adults with OUD,^28^ participants randomized to TAB achieved greater illicit opioid abstinence compared with control participants. Adherence to buprenorphine administration, daily monitoring, and random call-backs was high, and the iPad-delivered education produced significant improvements in HIV and HCV knowledge.^29^

Although those outcomes represented a positive first step, the RCT was limited to 12 weeks and conducted at a single, controlled university research clinic in Vermont’s only nonrural county. Although 30% of participants had previously overdosed (mean of 3.6 overdoses each), no overdose education was included. In the 2 concurrent RCTs presented here, we extended investigation of the TAB treatment to evaluate a longer duration, include overdose education, and address the rural communities especially affected by the opioid epidemic. Finally, although the practice of maintaining waiting lists has diminished in Vermont and many other areas in recent years, there remains a substantial number of individuals who are unable or unwilling to access treatment yet are not on a formal program waiting list. Thus, we also extended our investigation beyond solely individuals currently on a formal waiting list.

## Methods

### Trial Design and Setting

**Figure 1.**
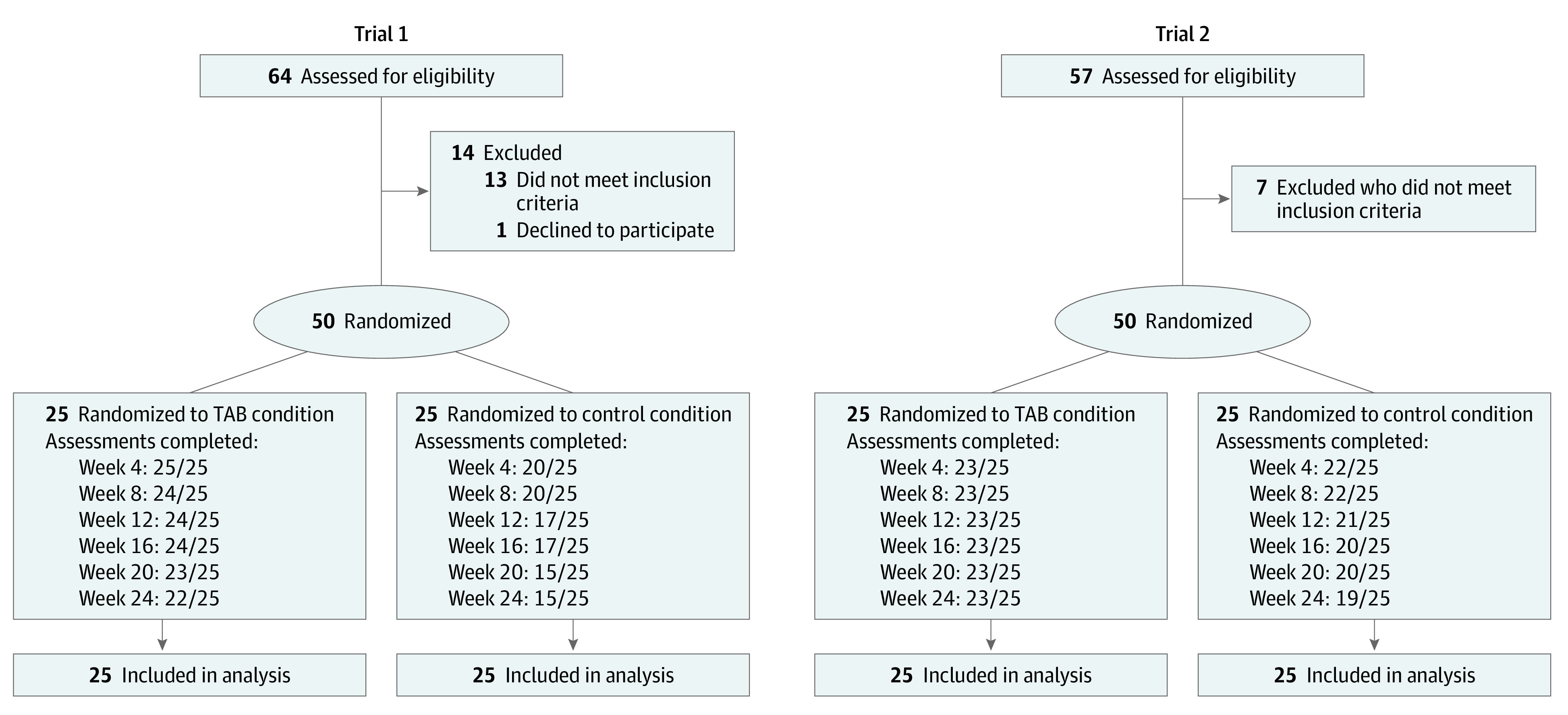
CONSORT Flow Diagrams Trial 1 participants were recruited from nonrural communities and trial 2 participants from rural communities. TAB indicates technology-assisted buprenorphine.

Trial protocols for these 2 RCTs were approved by the University of Vermont Institutional Review Board; participants provided written informed consent before participating. The primary research site was the University of Vermont Medical Center in Burlington. Participants were recruited via social media, community flyers, and referrals. Following an intake assessment determining eligibility, participants were randomized 1:1 to TAB or control conditions using minimum likelihood allocation to balance conditions on primary opioid used, amount of opioids used, lifetime intravenous opioid use, and waiting list status. After enrollment, participants completed 6 monthly assessments (weeks 4, 8, 12, 16, 20, and 24). Data collection was performed between February 1, 2018, and June 30, 2022. This report follows the Consolidated Standards of Reporting Trials (CONSORT) reporting guideline (Figure 1) with trial protocol details in Supplement 1.

Eligibility criteria, design, and methods were largely identical for both 24-week RCTs. The primary difference between the trials was the geographic areas from which participants were recruited. In trial 1, we evaluated the TAB protocol with its longer duration and new overdose education among individuals residing in the greater Burlington area, Vermont’s only urbanized area (≥50 000 population).^30^ Trial 1 visits took place at our research clinic. Trial 2 extended our investigation to individuals residing in rural communities (<50 000 population) farther from our research clinic. To reduce participant barriers, a mobile research team composed of a nurse and research assistant traveled to conduct visits at a convenient location in participants’ communities (eg, local fire department, municipal office, or community health center).

### Participants

Eligible participants for both trials had to be 18 years or older, meet *Diagnostic and Statistical Manual of Mental Disorders* (Fifth Edition)^31^ criteria for OUD, provide an opioid-positive urine test result, and not be currently receiving opioid agonist treatment. Those who were pregnant or breastfeeding or had a significant psychiatric or medical illness were excluded.^32^ Participants included 100 adults (50 per trial) who lived a mean (SD) of 4.23 (5.13) miles (trial 1) and 31.01 (12.42) miles (trial 2) from the research clinic.

### Measures

 Participants completed an intake screening. Participant characteristic data (eg, self-reported age, race and ethnicity, education level, and employment status) were collected as part of the clinic-developed drug history and demographic questionnaire. For demographic characterization of the samples, participants self-identified their race (selection of ≥1 of the following categories: African American or Black, American Indian or Alaska Native, Asian, Native Hawaiian or other Pacific Islander, White, or other) and ethnicity (Hispanic or Latinx: yes or no). Patients also completed the Beck Anxiety^33^ and Depression^34^ Inventories; *DSM-5* psychoactive substance use section^31^; HIV/AIDS,^35^ HCV,^36^ and overdose^37^ knowledge questionnaires; and medical history. Monthly assessments consisted of an abbreviated version of the intake, with an additional 6-item clinic-developed patient satisfaction survey at week 24. At all visits, participants’ past-month use of alcohol and prescribed and illicit drugs was assessed via timeline followback.^38^

### Biochemical Monitoring

Participants provided a staff-observed urine specimen at all visits, which was analyzed (Microgenics) for opioids (eg, methadone, buprenorphine, oxycodone, heroin, and fentanyl) and nonopioid drugs (eg, cocaine, amphetamines, and benzodiazepines). An opioid-positive specimen collected in the absence of a valid prescription was considered to be positive for illicit opioids. Failure to provide a scheduled specimen was considered a positive result for illicit opioids.

### TAB Condition

#### Buprenorphine Administration

Participants in the TAB group completed induction during week 1, receiving buprenorphine-naloxone sublingual tablets (Hikma Pharmaceuticals) under nurse observation with individualized dose adjustments using the Clinical Institute Narcotic Assessment.^39^ Thereafter, at 12 bimonthly visits, they ingested their dose under staff observation and provided a urine sample. Remaining doses during each 2-week interval were taken at home using a portable device during a predetermined 3-hour window (Med-O-Wheel Secure, Addoz). Participants brought the device to all scheduled and random call-back visits.

#### Daily Monitoring

Participants in the TAB group received prerecorded IVR calls each evening assessing opioid use, craving, and withdrawal as well as other drug or alcohol use.^23^ Reports of drug use prompted follow-up questions as well as encouragement to attend community meetings. The IVR calls also permitted immediate connection with staff if participants had urgent concerns or questions.

#### Random Call-Backs

Participants in the TAB group were contacted via IVR twice monthly on a random basis. During these calls, they were instructed to refrain from taking that day’s dose and instead return to the trial site within a specified time frame (typically 12 hours) to present their device for pill count, ingest that day’s dose under nurse observation, and provide a urine specimen.

#### HIV, HCV, and Overdose Education

Participants in the TAB group completed a baseline assessment (pretest) of HIV, HCV, and opioid overdose knowledge administered via iPad, with staff available to address questions or problems that arose. The HIV and HCV assessments provided immediate corrective feedback and explanations for incorrect items. Participants then reviewed an interactive flipbook,^40^ watched a video,^41^ and completed a brief overdose educational module.^37^ Assessments were readministered immediately after the session (posttest), after which staff offered harm reduction supplies and condoms. Participants completed the HIV, HCV, and overdose assessments again at weeks 4, 12, and 24.

### Control Condition

Control participants did not receive the medication or other services detailed above but completed the same monthly assessments with urinalysis. At each assessment, they received harm reduction supplies, condoms, a packet of community resources (eg, substance use treatment, mental health and medical care, and housing and employment resources) and assistance contacting any resources of interest.

### Statistical Analysis

Statistical analyses were conducted separately for trials 1 and 2. Primary analyses included all randomized participants consistent with an intention-to-treat approach.^42^ Repeated-measures analyses based on generalized estimating equation using a logit link function were used to compare TAB and control participants on the primary outcome of biochemically verified illicit opioid abstinence across monthly assessments. We used χ^2^ tests for group comparisons at each time point. Observed significance levels reflect a Bonferroni adjustment for multiple time points. A generalized estimating equation model was also used to examine abstinence across TAB participants’ biweekly visits. A linear contrast was constructed to test for a temporal trend. Mixed-model repeated-measure analyses were used to compare groups across monthly assessments on self-reported frequency of illicit opioid use and temporal changes in knowledge scores following HIV, HCV, and overdose education among TAB participants. The Bonferroni-adjusted Fisher least-significant difference procedure was used to compare scores at each time point with the baseline. Descriptive statistics were used to characterize TAB adherence and satisfaction. The sample size of 50 participants per RCT resulted in sufficient power (1 − β = 0.85) to detect group differences in abstinence of 40% (eg, 80% vs 40%) in the primary outcome. This estimated difference is conservative based on the earlier trial of shorter duration^28^ with an observed difference of greater than 65% at the 12-week assessment. A 2-sided *P* < .05 was considered statistically significant. Analyses were performed using SAS software, version 9.3 (SAS Institute Inc).

## Results

### Participants

#### Trial 1

The 50 nonrural participants had a mean (SD) age of 40.6 (13.1) years; 28 (56.0%) were male and 22 (44.0%) were female; 1 (2.0%) was African American or Black; 4 (8.0%), American Indian; 1 (2.0%), Hispanic or Latinx; 1 (2.0%), multiracial; and 43 (86.0%), White (Table 1). Participants reported using opioids regularly for a mean (SD) of 9.4 (7.6) years, and 27 (54.0%) reported a lifetime history of intravenous opioid use. Although most endorsed a prescription opioid as their current primary opioid, 32 (64.0%) reported a lifetime history of heroin use. Fifteen (30.0%) had overdosed from opioids in the past. The TAB participants’ mean (SD) buprenorphine dose was 9.1 (4.0) mg.

**Table 1.** Baseline Demographic Characteristics by Group in Trials 1 and 2^a^

Characteristic	Trial 1			Trial 2		
	Both groups (n = 50)	TAB (n = 25)	Control (n = 25)	Both groups (n = 50)	TAB (n = 25)	Control (n = 25)
Age, mean (SD), y	40.6 (13.1)	40.5 (12.9)	40.7 (13.2)	40.3 (10.8)	39.3 (10.0)	41.3 (11.5)
Sex						
Female	22 (44.0)	10 (40.0)	12 (48.0)	20 (40.0)	7 (28.0)	13 (52.0)
Male	28 (56.0)	15 (60.0)	13 (52.0)	30 (60.0)	18 (72.0)	12 (48.0)
Race and ethnicity						
African American or Black	1 (2.0)	0	1 (4.0)	0	0	0
American Indian	4 (8.0)	1 (4.0)	3 (12.0)	3 (6.0)	2 (8.0)	1 (4.0)
Hispanic or Latinx	1 (2.0)	1 (4.0)	0	1 (2.0)	0	1 (4.0)
Multiracial	1 (2.0)	1 (4.0)	0	1 (2.0)	0	1 (4.0)
White	43 (86.0)	22 (88.0)	21 (84.0)	45 (90.0)	23 (92.0)	22 (88.0)
Employed full time	20 (40.0)	10 (40.0)	10 (40.0)	26 (52.0)	17 (68.0)	9 (36.0)
Education level, mean (SD), y	12.4 (1.5)	12.2 (1.6)	12.6 (1.3)	12.4 (1.8)	12.8 (2.1)	12.0 (1.5)
Duration of regular opioid use, mean (SD), y	9.4 (7.6)	9.3 (6.6)	9.5 (8.5)	9.5 (6.1)	9.2 (6.2)	9.8 (5.9)
Past-month illicit opioid use, mean (SD), d	25.9 (5.3)	26.7 (4.5)	25.1 (6.1)	27.6 (4.9)	27.1 (4.5)	28.1 (5.2)
Ever used intravenous route for opioids	27 (54.0)	14 (56.0)	13 (52.0)	22 (44.0)	10 (40.0)	12 (48.0)
Ever used heroin	32 (64.0)	17 (68.0)	15 (60.0)	33 (66.0)	16 (64.0)	17 (68.0)
Ever overdosed on opioids	15 (30.0)	6 (24.0)	9 (36.0)	13 (26.0)	5 (20.0)	7 (28.0)
Any past-month cocaine use	19 (38.0)	10 (40.0)	9 (36.0)	13 (26.0)	7 (28.0)	6 (24.0)
Past year primary opioid of abuse						
Prescription opioid	39 (78.0)	19 (76.0)	20 (80.0)	46 (92.0)	23 (92.0)	23 (92.0)
Buprenorphine	26 (52.0)	12 (48.0)	14 (56.0)	30 (60.0)	15 (60.0)	15 (60.0)
Oxycodone	6 (12.0)	4 (16.0)	2 (8.0)	10 (20.0)	5 (20.0)	5 (20.0)
Hydrocodone	3 (6.0)	1 (4.0)	2 (8.0)	3 (6.0)	2 (8.0)	1 (4.0)
Hydromorphone	2 (4.0)	1 (4.0)	1 (4.0)	1 (2.0)	0	1 (4.0)
Morphine	1 (2.0)	1 (4.0)	0	2 (4.0)	1 (4.0)	1 (4.0)
Methadone	1 (2.0)	1 (4.0)	0	0	0	0
Heroin	10 (20.0)	5 (20.0)	5 (20.0)	4 (8.0)	2 (8.0)	2 (8.0)
Fentanyl	1 (4.0)	1 (4.0)	0	0	0	0
Primary route of opioid administration						
Oral or sublingual	25 (50.0)	14 (56.0)	11 (44.0)	34 (68.0)	14 (56.0)	20 (80.0)
Intranasal	15 (30.0)	8 (32.0)	7 (24.0)	11 (22.0)	8 (32.0)	3 (12.0)
Intravenous	11 (22.0)	3 (12.0)	8 (32.0)	4 (8.0)	3 (12.0)	1 (4.0)
Inhalation	0	0	0	1 (2.0)	0	1 (4.0)
Beck Anxiety Inventory score, mean (SD)^b^	10.1 (9.2)	9.6 (9.7)	10.5 (8.7)	6.8 (8.6)	4.5 (4.8)	9.0 (12.3)
Beck Depression Inventory score, mean (SD)^c^	16.6 (12.6)	16.4 (12.2)	16.7 (12.9)	13.1 (11.3)	10.0 (7.0)	16.2 (15.6)
Michigan Alcohol Screening Test score, mean (SD)^d^	10.1 (10.4)	10.4 (10.7)	9.8 (10.0)	7.3 (8.0)	7.1 (7.5)	7.5 (8.5)
Chronic pain	26 (54.2)	13 (54.2)	13 (54.2)	23 (46.0)	13 (56.5)	10 (40.0)
Buprenorphine dose, mean (SD), mg	NA	9.1 (4.0)	NA	NA	10.7 (3.6)	NA

Abbreviation: NA, not applicable.

^a^ Data are presented as number (percentage) of study participants unless otherwise indicated.

^b^ The Beck Anxiety Inventory measures the intensity of anxiety experienced during the past week. Scores of 0 to 7 indicate minimal anxiety; 8 to 15, mild anxiety; 16 to 25, moderate anxiety; and 26 to 63, severe anxiety.

^c^ The Beck Depression Inventory measures the intensity of depressive symptoms during the past 2 weeks. Scores of 0 to 13 indicate minimal depression; 14 to 19, mild depression; 20 to 28, moderate depression; and 29 to 63, severe depression.

^d^ The Michigan Alcohol Screening Test is a 25-question test to help identify alcohol dependency. Scoring 5 points or more indicates the probability of substance abuse; 4 points is suggestive; and 3 points is normal. Scoring 8 points or above is strong evidence for chronic abuse or dependence.

#### Trial 2

Rural participants’ baseline characteristics were similar to the trial 1 sample (mean [SD] age, 40.3 [10.8] years; 30 [60.0%] male and 20 [40.0%] female; 0 African American or Black, 3 [6.0%] American Indian, 1 [2.0%] Hispanic or Latinx, 1 [2.0%] multiracial, and 45 [90.0%] White). Participants reported using opioids for a mean (SD) of 9.5 (6.1) years, and 22 (44.0%) reported lifetime intravenous opioid use. Although prescription opioids were their primary opioid, 33 (66.0%) reported lifetime heroin use and 13 (26.0%) reported a past opioid overdose. More TAB participants were employed full-time than controls (17 [68.0%] vs 9 [36.0%]). The TAB participants’ mean (SD) buprenorphine dose was 10.7 (3.6) mg.

### Biochemically Verified Illicit Opioid Abstinence 

#### Trial 1

**Figure 2.**
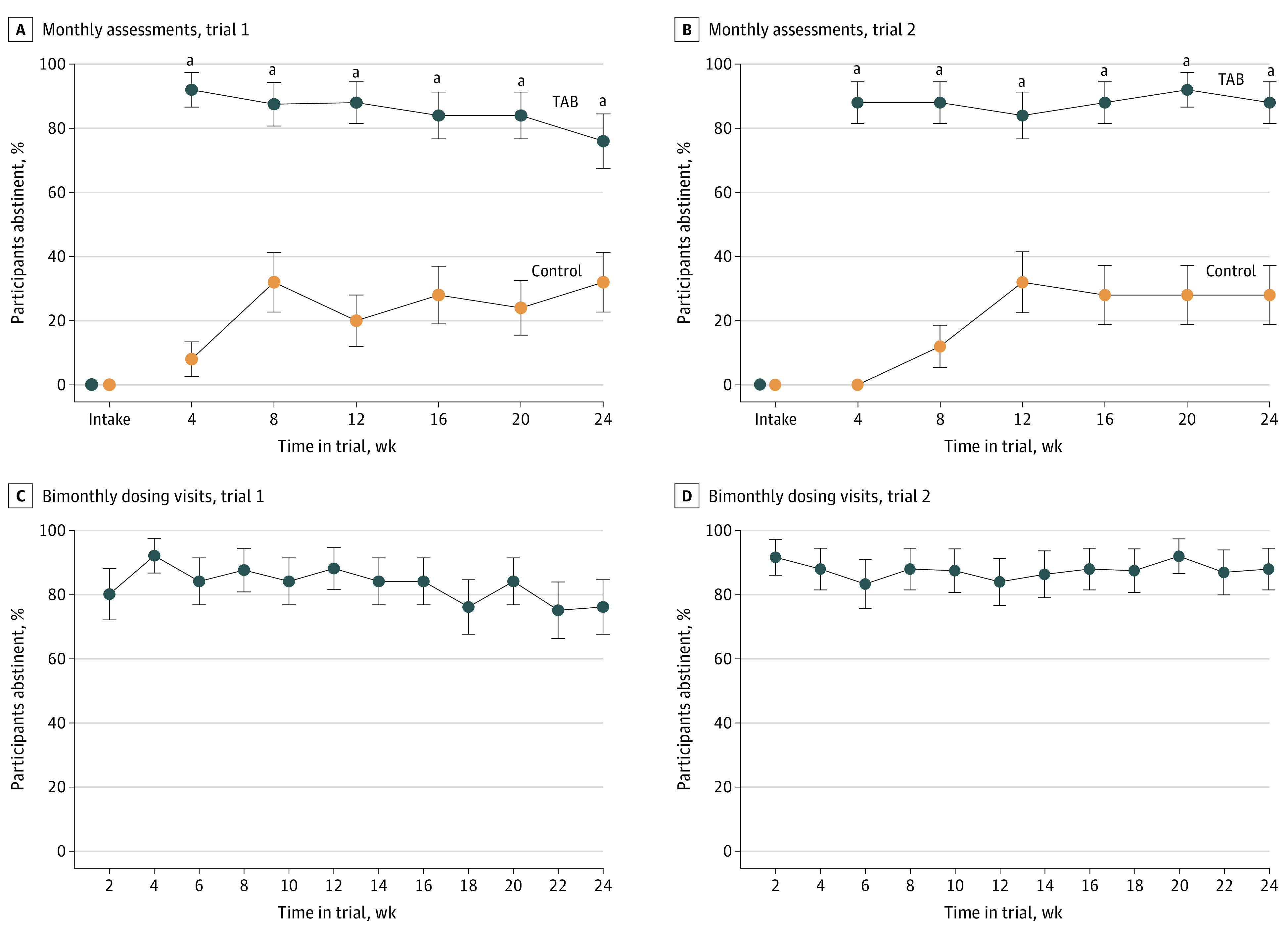
Illicit Opioid Abstinence Error bars indicate SEMs. TAB indicates technology-assisted buprenorphine. ^a^Significant difference between conditions using Bonferroni-adjusted χ^2^ tests (*P* < .05).

Overall, illicit opioid abstinence across all monthly assessments was 85.3% (128 of 150; 95% CI, 70.7%-93.3%) and 24.0% (36 of 150; 95% CI, 13.6%-38.8%) for the TAB and control conditions, respectively (difference, 61.3%; 95% CI, 44.6%-78.1%; *P* < .001). The percentage of participants abstinent from illicit opioids was significantly greater for TAB participants than controls at all time points (Table 2 and Figure 2A). Participants in the TAB and control conditions both submitted urine specimens at 97.0% (TAB: 97.2% [138 of 142]; control: 97.1% [101 of 104]) of scheduled monthly assessments while retained.

**Table 2.** Primary and Secondary Outcomes for Trials 1 and 2

Outcome measure	Trial 1				Trial 2			
	TAB (n = 25)	Control (n = 25)	Difference (95% CI)	*P* value	TAB (n = 25)	Control (n = 25)	Difference (95% CI)	*P* value
**Illicit opioid abstinence, No. (%)**								
Intake	0	0	NA^a^	NA^a^	0	0	NA^a^	NA^a^
Week 4	23 (92)	2 (8)	84 (69 to 99)^b^	<.001^c^	22 (88)	0	88 (75 to 99)^b^	<.001^c^
Week 8	22 (88)	8 (32)	56 (34 to 78)^b^	<.001^c^	22 (88)	3 (12)	76 (58 to 94)^b^	<.001^c^
Week 12	22 (88)	5 (20)	68 (48 to 88)^b^	<.001^c^	21 (84)	8 (32)	52 (29 to 75)^b^	.001^c^
Week 16	21 (84)	7 (28)	56 (33 to 79)^b^	<.001^c^	22 (88)	7 (28)	60 (38 to 82)^b^	<.001^c^
Week 20	21 (84)	6 (24)	60 (38 to 82)^b^	<.001^c^	23 (92)	7 (28)	64 (43 to 85)^b^	<.001^c^
Week 24	19 (76)	8 (32)	44 (19 to 69)^b^	.01^c^	22 (88)	7 (28)	60 (38 to 82)^b^	<.001^c^
**Self-reported past-month illicit opioid use, mean (SD), d**								
Intake	26.7 (4.5)	25.1 (6.1)	NA^d^	NA^d^	27.1 (4.5)	28.1 (5.2)	NA^d^	NA^d^
Week 4	0.6 (1.1)	21.5 (11.0)	−20.9 (−25.7 to −16.1)	<.001^e^	1.2 (2.1)	27.4 (6.0)	−26.2 (−30.9 to −21.6)	<.001^e^
Week 8	0.6 (1.8)	16.9 (11.2)	−16.3 (−21.1 to −11.4)	<.001^e^	0.4 (0.7)	22.3 (11.1)	−21.9 (−26.6 to −17.2)	<.001^e^
Week 12	0.9 (3.1)	13.3 (12.0)	−12.4 (−17.4 to −7.4)	<.001^e^	0.5 (0.7)	19.1 (12.6)	−18.6 (−23.3 to −13.9)	<.001^e^
Week 16	2.4 (6.7)	13.4 (13.1)	−11.0 (−15.9 to −6.1)	<.001^e^	0.8 (2.3)	16.2 (14.3)	−15.4 (−20.1 to −10.6)	<.001^e^
Week 20	2.1 (6.7)	11.5 (13.0)	−9.4 (−14.4 to −4.3)	.002^e^	0.4 (0.8)	16.5 (14.4)	−16.1 (−20.9 to −11.3)	<.001^e^
Week 24	1.1 (3.1)	10.5 (13.2)	−9.4 (−14.5 to −4.2)	.002^e^	0.5 (1.0)	18.3 (13.9)	−17.8 (−22.6 to −13.1)	<.001^e^
**HIV knowledge, mean (SD), % correct**								
Intake	57.9 (15.0)	NA^f^	NA^f^	NA^f^	61.6 (15.2)	NA^f^	NA^f^	NA^f^
Posttest	77.7 (18.5)	NA^f^	19.8 (14.3 to 25.4)	<.001^g^	79.9 (15.1)	NA^f^	18.3 (13.7 to 22.9)	<.001^g^
Week 4	74.8 (23.7)	NA^f^	16.9 (11.2 to 22.6)	<.001^g^	77.7 (19.5)	NA^f^	16.1 (11.3 to 20.8)	<.001^g^
Week 12	74.7 (18.6)	NA^f^	16.8 (11.1 to 22.6)	<.001^g^	80.0 (10.9)	NA^f^	18.4 (13.4 to 23.4)	<.001^g^
Week 24	74.4 (19.2)	NA^f^	16.5 (10.7 to 22.3)	<.001^g^	80.7 (12.9)	NA^f^	19.1 (14.4 to 23.8)	<.001^g^
**HCV knowledge, mean (SD), % correct**								
Intake	56.7 (18.8)	NA^f^	NA^f^	NA^f^	50.4 (27.8)	NA^f^	NA^f^	NA^f^
Posttest	81.2 (16.1)	NA^f^	24.5 (18.4 to 30.6)	<.001^g^	78.7 (20.8)	NA^f^	28.3 (21.9 to 34.7)	<.001^g^
Week 4	67.9 (24.2)	NA^f^	11.2 (5.0 to 17.4)	.005^g^	74.1 (20.6)	NA^f^	23.7 (17.2 to 30.3)	<.001^g^
Week 12	71.4 (19.9)	NA^f^	14.7 (8.5 to 21.0)	<.001	80.0 (14.1)	NA^f^	29.6 (22.7 to 36.5)	<.001^g^
Week 24	70.2 (25.8)	NA^f^	13.5 (7.2 to 19.7)	<.001^g^	75.3 (22.2)	NA^f^	24.9 (18.4 to 31.4)	<.001^g^
**Overdose knowledge, mean (SD), % correct**								
Intake	76.0 (18.1)	NA^f^	NA^f^	NA^f^	71.7 (19.4)	NA^f^	NA^f^	NA^f^
Posttest	92.7 (9.4)	NA^f^	16.7 (9.6 to 23.7)	<.001^g^	92.2 (7.5)	NA^f^	20.5 (13.7 to 27.3)	<.001^g^
Week 4	91.3 (20.8)	NA^f^	15.3 (8.1 to 22.5)	<.001^g^	87.6 (22.6)	NA^f^	15.9 (8.9 to 22.9)	<.001^g^
Week 12	86.1 (16.4)	NA^f^	10.1 (2.8 to 17.4)	.03^g^	92.3 (9.7)	NA^f^	20.6 (13.2 to 27.9)	<.001^g^
Week 24	87.1 (23.0)	NA^f^	11.1 (3.8 to 18.4)	.01^g^	92.3 (9.3)	NA^f^	20.6 (13.7 to 27.5)	<.001^g^

Abbreviations: HCV, hepatitis C virus; NA, not applicable; TAB, technology-assisted buprenorphine.

^a^ All participants tested positive for opioids at intake per inclusion criterion.

^b^ For illicit opioid abstinence, the difference (95% CI) values are the differences in percentages.

^c^ *P* value corresponds to testing null hypothesis of equality of percentages between TAB and control participants at each assessment based on Bonferroni-adjusted χ^2^ tests.

^d^ Participants were randomized to treatment arms; hypothesis testing not appropriate at intake assessment.

^e^ *P* value corresponds to testing null hypothesis of equality of means between TAB and control participants at each assessment based on Bonferroni-adjusted Fisher least-significant difference test.

^f^ Control participants did not receive HIV, HCV, and overdose knowledge education intervention.

^g^ *P* value corresponds to testing null hypothesis of no change in mean score from intake to each assessment in TAB participants only based on Bonferroni-adjusted Fisher least-significant difference test.

#### Trial 2

Similar outcomes were seen in trial 2. Abstinence across all monthly assessments was 88.0% (132 of 150; 95% CI, 72.1%-95.4%] and 21.3% (32 of 150; 95% CI, 11.4%-36.5%) for the TAB and control conditions, respectively (difference, 66.7%; 95% CI, 49.9%-83.4%; *P* < .001). The percentage of participants abstinent from illicit opioids was significantly greater for TAB participants than controls at all time points (Table 2 and Figure 2B). The TAB and control participants submitted urine samples at 100% (138 of 138) and 96.8% (119 of 123) of monthly assessments while retained, respectively.

### Additional Illicit Opioid Abstinence Outcomes

#### Trial 1

At TAB participants’ bimonthly dosing visits, 83.0% (249 of 300; 95% CI, 67.0%-92.0%) of specimens tested negative for illicit opioids with no significant decrease over time (Table 2 and Figure 2C). The TAB participants also reported a significantly lower frequency of illicit opioid use than controls at all postintake time points (Table 2).

#### Trial 2

At TAB participants’ bimonthly visits, 88.0% (264 of 300; 95% CI, 71.0%-95.0%) of specimens tested negative for illicit opioids with no significant decrease over time (Table 2 and Figure 2D). Also consistent with trial 1, TAB participants reported a significantly lower frequency of illicit opioid use than controls at all time points (Table 2).

### TAB Treatment Adherence and Satisfaction

#### Trial 1

Among TAB participants, 98.9% (3614 of 3653) of buprenorphine doses were taken as scheduled. These participants completed 92.9% (3375 of 3632) of daily IVR calls, with a mean (SD) duration of 1.1 (0.4) minutes. Participants completed 91.7% (187 of 204) of scheduled random call-backs, with 97.9% (183 of 187) of specimens collected at those visits testing negative for illicit opioids. At week 24, TAB participants rated their satisfaction with treatment as a whole and its individual components from 1 (not at all) to 5 (extremely). Mean ratings were 4.9 (95% CI, 4.6-5.0) for the full treatment and 4.8 (95% CI, 4.5-5.0) for buprenorphine, 3.9 (95% CI, 3.4-4.5) for the portable device, 3.3 (95% CI, 2.7-3.8) for IVR, and 3.8 (95% CI, 3.1-4.4) for random call-backs. When asked to identify the most valuable treatment component, 18 of 23 participants (78.3%) endorsed buprenorphine.

#### Trial 2

Similar outcomes were seen in trial 2, with 99.3% (3812 of 3840) of doses taken as scheduled and 92.5% (3558 of 3848) of IVR calls completed, with a mean (SD) duration of 1.2 (0.4) minutes. These participants completed 91.8% (180 of 196) of random call-backs, with 97.8% (176 of 180) of specimens testing negative for illicit opioids. Participants’ satisfaction ratings were 4.9 (95% CI, 4.7-5.0) for the full treatment and 4.9 (95% CI, 4.7-5.0) for buprenorphine, 4.1 (95% CI, 3.6-4.6) for the portable device, 2.6 (95% CI, 2.0-3.1) for the IVR, and 3.3 (95% CI, 2.7-3.8) for random call-backs. When asked to identify the most valuable component, 19 of 23 (82.6%) endorsed buprenorphine.

### HIV, HCV, and Opioid Overdose Education

#### Trial 1

**Figure 3.**
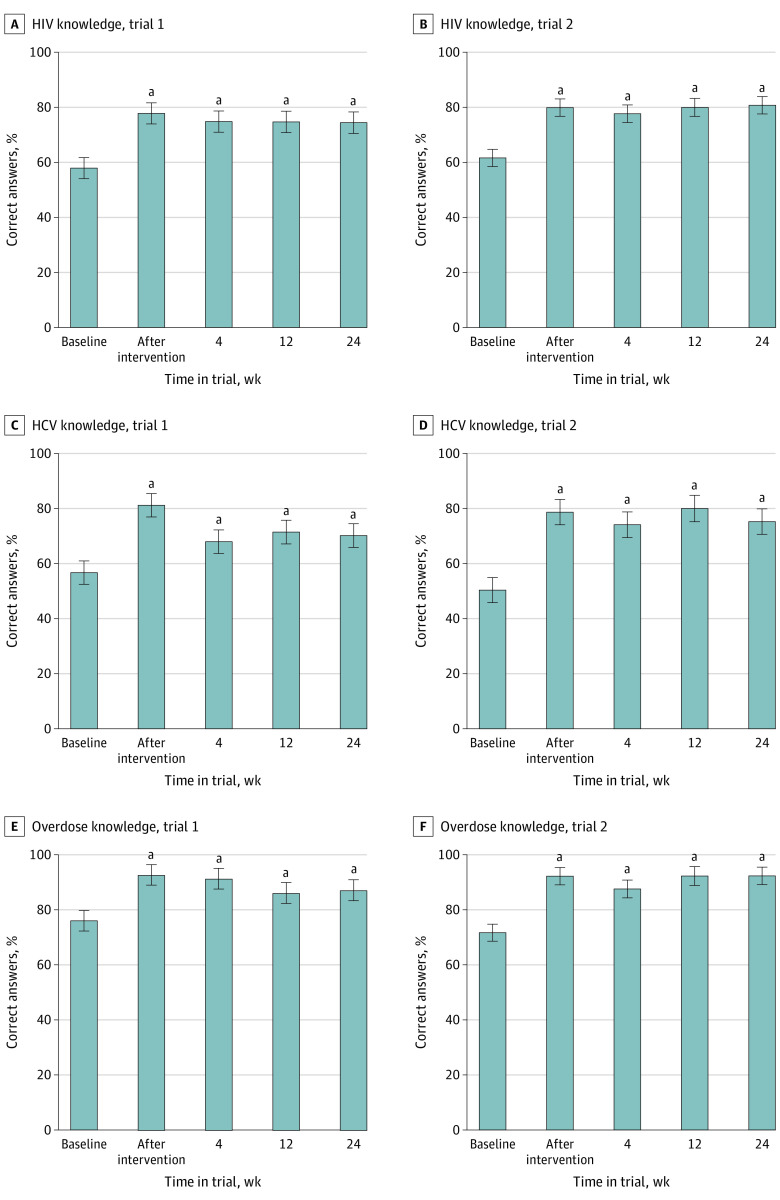
HIV, Hepatitis C Virus (HCV), and Opioid Overdose Knowledge Mean percentage of items answered correctly on the HIV, HCV, and overdose knowledge assessments are presented for technology-assisted buprenorphine participants in trial 1 and trial 2. For both studies, data are presented from the baseline knowledge assessment (pretest), the assessment immediately after the educational intervention (posttest), and the week 4, 12, and 24 assessments. Error bars indicate SEMs. ^a^Significantly different from baseline assessment (pretest) using Bonferroni-adjusted Fisher least-significant difference test (*P* < .05).

On assessments completed by TAB participants (pretest), a mean (SD) of 57.9% (15.0%) of HIV items, 56.7% (18.8%) of HCV items, and 76.0% (18.1%) of overdose baseline knowledge items were answered correctly (Table 2 and Figure 3). Significant improvements were observed on all 3 assessments immediately after the educational session. These improvements persisted throughout the study, with mean scores on all assessments at all time points greater than baseline.

#### Trial 2

Similarly, trial 2 TAB participants answered a mean (SD) of 61.6% (12.2%) of HIV items, 50.4% (27.8%) of HCV items, and 71.7% (19.4%) of overdose baseline knowledge items correctly (Table 2 and Figure 3). Scores on all 3 assessments were significantly greater after the session, and the improvements were sustained, with all scores greater than baseline.

## Discussion

These data extended prior work^28^ by evaluating TAB efficacy for a longer duration, which was important given the nonsignificant decrease in abstinence observed toward the end of that 3-month pilot RCT. The sustained abstinence during the full 24-week duration suggested stability of treatment effects, although still longer evaluations will be helpful for understanding effects for the extended periods typical of buprenorphine maintenance. Also worth noting are abstinence levels of greater than 20% among control participants by the end of these studies, which differs from the 0% in the pilot trial.^28^ As noted above, control participants were not prohibited from seeking treatment elsewhere and in fact at each visit received community resources and assistance contacting any practitioners of interest. The gradual increases in abstinence as the study progressed were generally a function of some participants entering treatment elsewhere because treatment availability in Vermont markedly improved between the time of the pilot and current trials.

Adherence to TAB treatment was favorable and nearly identical between the trials, with rates of approximately 99% for buprenorphine administration, 93% for daily IVR calls, and 92% for random call-backs. This was similar to the adherence seen in the pilot^28^ (99%, 96%, and 96%, respectively) despite the present trials being twice as long. Higher rates of follow-up assessment completion were seen in TAB participants, likely because of their receiving active treatment (ie, buprenorphine) as part of the trial. Participant ratings of satisfaction were also high and consistent with the pilot trial (ie, 4.9 and 4.6, respectively, on a 5-point scale), although future studies could explore ways to improve satisfaction with the IVR component, which had the lowest rating.

The iPad-delivered HIV and HCV modules were associated with significant knowledge increases of a similar magnitude to the pilot study.^29^ These improvements were sustained in the 24 weeks after the intervention, without additional education or booster sessions. Our addition of the overdose assessment and education^37,43,44^ represented a novel extension from earlier work. As with the HIV and HCV education, the overdose module was associated with significant and sustained knowledge improvements in both studies, providing additional support that mHealth applications can improve knowledge among high-risk populations.^44,45,46^

Finally, these data extended support for TAB efficacy to rural settings, with consistent treatment effects across nonrural and rural samples. Our successful establishment of remote sites highlights the likely value of partnerships in rural communities to facilitate OUD treatment delivery. The mHealth components themselves also hold potential for use with patients who are unable or unwilling to access in-person treatment options, as well as other contexts where adherence support may be beneficial.

### Limitations

Several limitations merit mention. First, we did not empirically evaluate the potential impact of the 2 modifications made to facilitate delivery with rural participants (ie, remote sites and mobile treatment team). Subsequent efforts could experimentally isolate the contribution of these components to treatment effects. Second, we did not examine the effects of HIV, HCV, and overdose education in control participants or on TAB participants’ behavior, although prior reports have demonstrated a strong concordance between education and risk behavior reductions.^47,48,49,50^ Third, although these 2 trials extended our evaluation of this intervention for a longer duration and to patients residing in rural communities, sample sizes were still limited, and we did not experimentally isolate the contribution of individual treatment components. Future studies could examine these issues as well as how TAB may be adapted for nonresearch settings. Fourth, although the inclusion criterion used in the initial pilot study^28^ that individuals must currently be on a formal waiting list was relaxed for these trials, our findings were consistent with those earlier results and suggest that treatment effects are not specific to an individual’s waiting list status.

## Conclusions

In these RCTs of TAB treatment, demonstration of efficacy was extended to patients residing in rural communities and to a longer duration than previously examined. To the extent that its components supported adherence to treatment, this protocol may be useful with patients who need additional clinical support or for those who are unable to regularly attend treatment visits because of pragmatic barriers (eg, geographic distance and lack of reliable transportation or childcare). Reductions in practitioner burden and concerns about diversion risk could improve physicians’ willingness to prescribe. Overall, use of these and other mHealth platforms may help expand much-needed treatment capacity in underserved areas and populations.

## Notes

Supplement 1.
Trial Protocol

Supplement 2.
Data Sharing Statement
