# Supplementary material for: Technology-Assisted Buprenorphine Treatment in Rural and Nonrural Settings: Two Randomized Clinical Trials
Source: JAMA Netw Open. 2023 Sep 27;6(9):e2331910. doi: 10.1001/jamanetworkopen.2023.31910 (PMC10534272; doi:10.1001/jamanetworkopen.2023.31910)
Supplement: Supplement 1. — Trial Protocol [file jamanetwopen-e2331910-s001.pdf]

The University of Vermont Committees on Human Research

For Committee Use Only PROTOCOL NUMBER

Human Subjects Research Protocol

The Common Human Subjects Protocol Cover Form **must** be completed and **attached** to the front of this form. This Protocol form should be completed for any human subjects research proposal that does not have a specific "protocol," such as a grant application. This form must be submitted along with a copy of the complete grant proposal and all the information in this form **must** be consistent with that proposal. This protocol form, once IRB approved, will be the working protocol for that research. **When completing this document, do not refer to page numbers within your grant**. If revisions are necessary during the course of the research, amendments should refer to this protocol form, not the grant proposal. Enter responses for all sections. Check N/A if the section does not apply.

PROTOCOL SUMMARY

Project Title:	Protocol Version Date:
Interim Buprenorphine Treatment to reduce individual and societal harm during delays to opioid treatment: Extending IBT duration (Trial 1)	10/10/17

Principal Investigator: Stacey C. Sigmon

Grant Sponsor: Laura and John Arnold Foundation Grant Number: TBD
(For grants routed through UVM, indicate the OSP Proposal ID # located at the top of the OSP Routing Form)

PURPOSE AND OBJECTIVES

Purpose: *The importance of the research and the potential knowledge to be gained should be explained in detail. Give background information.*

Opioid abuse and dependence have reached epidemic proportions in the US, resulting in premature death, criminal activity, and economic costs of over $56 billion annually (Becker et al., 2008; Birnbaum et al., 2011; Clausen et al., 2009; Jones et al., 2013; Shah et al., 2008; SAMHSA, 2010; Wisniewski et al., 2008). The recent increases in opioid-related overdoses are particularly concerning, with 3- and 6-fold increases in overdose deaths related to prescription opioids and heroin, respectively, between 2001 and 2014 (CDC, 2015). Untreated opioid dependence has been associated with unprecedented outbreaks of HIV and Hepatitis C (Strathdee & Beyrer, 2015), and overdose deaths have now surpassed motor vehicle fatalities to become the country's leading cause of injury death (CDC, 2016).

Treatment with agonist medications (e.g., methadone, buprenorphine) represents the most efficacious treatment for opioid dependence and dramatically reduces morbidity, mortality and spread of infectious disease (Schwartz et al., 2013; Stotts et al., 2009). However, demand for treatment far exceeds available capacity in many areas of the country (Friedmann et al., 2003; Harlow et al., 2013; Sigmon, 2014), with ***96% of states in the US having opioid abuse rates that exceed their medication-assisted treatment capacity*** (Jones et al., 2015). The issue of insufficient treatment availability is especially urgent in rural areas (Paulozzi & Xi, 2008; Rintoul et al., 2011; Rosenblum et al., 2011; Sigmon, 2014; Stein et al., 2015). The end result is that opioid-dependent individuals can remain on waitlists for months, during which they are at significant risk for illicit drug use, criminal activity, infectious disease, overdose and mortality (Chun et al., 2008; Clausen et al., 2009; Schwartz et al., 2009b). In one evaluation of survival rates among opioid-dependent individuals who received waitlist vs. immediate entry into methadone treatment, for example, ***opioid treatment waitlists were associated with a 12-fold increase in risk of premature death*** (Peles et al., 2013).

One effort to mitigate individual and societal risks during treatment delays has been to offer interim methadone treatment (IMT; i.e., daily methadone without counseling) to individuals awaiting entry into a methadone program (Sigmon, 2015b). IMT has been shown to produce dramatic reductions in drug use, risk behaviors and criminal activity during treatment delays (Gruber et al., 2008; Schwartz et al., 2006, 2011; Yancovitz et al., 1991). However, few clinics have implemented this approach, likely due to

methadone's regulatory and pharmacological limitations. Methadone treatment is limited to licensed specialty clinics and the medication itself has risks of diversion, abuse and overdose with misuse (Luty et al., 2005). IMT regulations also limit its duration to no more than 120 days, with clinics required to admit patients at that time or discharge them if a treatment slot is still not available. Importantly, they also prohibit take-home doses and require that patients ingest all doses under direct staff observation, resulting in daily clinic visits. These features are incompatible with an environment of constrained resources and have limited the widespread adoption of IMT.

We have developed a novel **Interim Buprenorphine Treatment (IBT)**, aimed at reducing the risks associated with treatment delays while surmounting the limitations noted above for methadone. The intervention includes several key components to permit delivery of life-saving pharmacotherapy while minimizing nonadherence:

(a) The partial opioid agonist buprenorphine (BUP) has a favorable abuse liability and safety profile and it attenuates the effects of other opioids, thereby suppressing illicit use during treatment (Johnson et al., 2003). Importantly, it is also available without the rigid regulations, daily dosing requirements, and 120-day interim-dosing limit required for methadone, making it uniquely compatible with an interim-dosing approach for opioid dependence. Our IBT intervention consists of BUP maintenance with participants' visiting the research clinic bi-monthly to ingest that days dose under nurse observation and then receiving the remaining doses for ingestion at home via a secure computerized portable device (Med-O-Wheel Secure, Addoz, Forssa; Fig 1). The Med-O-Wheel is a portable, tamper resistant pill dispenser. Each day's dose is secured in separate compartments around the cassette and available for a 3-hour window which is programmed by staff. At the specified dosing time, an alarm sounds and the user presses a button on the device cover. The Med-O-Wheel moves the correct dose into place for release. Otherwise the device remains locked until the next specified dosage time and all medication is secured inside the unit. It also includes a visual alarm (i.e., red light on the bottom of the unit) that is activated if there is an attempt to tamper with the device or access to medication outside the preset time window.

(b) An intensive clinical support package is impractical for the resource-constrained settings in which IBT is likely to be delivered. However, one alternative is the rapidly-expanding use of mobile health (*m*Health) platforms, particularly given the increasing number of portable devices with sophisticated functionality (Boyer et al., 2010). *m*Health platforms can extend the reach of health care by permitting delivery of monitoring, education, point-of-care diagnostics and treatment beyond the confines of the medical office. Interactive Voice Response (IVR) systems are particularly promising in that they can provide customized content and support via phone and offer advantages of low cost, consistent delivery, expanded access, 24-hour availability and convenience (Helzer et al., 2008; Rose et al., 2010ab; Stacy et al., 2009). Patients use keypad or voice to respond to prompts and answer questions.

IVR is an ideal choice for IBT. Phone-based systems are compatible with resource-constrained settings as they require no specialized equipment or extensive training. IVR hardware and software can support multiple clinic sites and have no on-site installation costs beyond telephone access. They provide broad access for marginalized populations, as phones are familiar, easy to use, and more widely available than computers. IVR uses an auditory interactive process that is not hampered by low literacy. Privacy is also greater than on a computer screen or written questionnaire. We have developed an IVR system that is compatible with the low-intensity, extended-reach IBT approach. It calls participants nightly to assess any opioid or other drug use, as well as opioid craving and withdrawal. It includes information about self-help meetings in the community and immediate connection with research staff if needed. To maximize participant convenience and adherence, we designed the system to automatically call participants each day rather than requiring them to call into the system; however, it also accepts inbound calls if the participant prefers or anticipates missing the incoming call.

(c) Biochemical verification, typically via urine toxicology, is the most objective method for evaluating recent drug use (Chermack et al., 2000; Fendrich et al., 2004; Kilpatrick et al., 2000; Preston et al., 1997; Wish et al., 1997). Our long-standing protocol involves thrice-weekly urinalysis monitoring during the early months of treatment, followed by a reduction to twice weekly once participants are stable (Higgins, Sigmon et al., 2003). While this rigorous protocol maximizes detection of even low levels of drug use (Cone & Dickerson, 1992), thrice-weekly visits are incompatible with IBT and resource-constrained settings more generally. To balance the rigor of the above procedure with the less-intensive schedule necessary for IBT, we developed a random call-back protocol wherein patients are contacted by IVR at random times and instructed to return to the clinic. Random sampling increases the effectiveness of monitoring, as patients are always in the position of not knowing when the next drug screen will be requested, reducing the

possibility that s/he can tailor drug use to subvert monitoring (Harford & Kleber, 1978; Manno, 1986). For each random call-back, the IVR system contacts participants and instructs them to visit the clinic to provide a staff-observed urine specimen and present their Med-O-Wheel for inspection to ensure there is no evidence of tampering or nonadherence.

(d) Finally, while IBT is intended as a low-intensity, extended-reach paradigm, we believe it is essential to include an intervention to enhance HIV and hepatitis knowledge in this extremely high-risk group. HIV and hepatitis (HCV) are responsible for an estimated 30,000 US deaths annually, and deaths from HCV-related liver complications are increasing (CDC, 2008, 2011; Larney et al., 2012). Brief psychosocial interventions that focus on increasing knowledge about disease prevention are appealing in that they can be done in a single session, do not require extensive counselor training, and have been associated with decreases in self-reported risk behaviors (Copenhaver et al., 2006; Meader et al., 2010).

Our team has developed a single-visit intervention that produces significant improvements in HIV and HCV knowledge in drug-dependent adults (Dunn et al., 2013; Heil et al., 2005; Herrmann et al., 2013). Participants complete a baseline knowledge assessment (Pre-Test) followed by corrective feedback, both administered via iPad. They then complete an interactive HIV flipbook (Fig 2) and animated HCV video, followed by a repeat administration of the knowledge assessment (Post-Test). Finally, to evaluate whether improvements in knowledge persist in the months following the educational intervention, the Post-Test is administered again at 4 and 12 weeks following intake.

Supporting preliminary data. We recently completed a small pilot study evaluating the initial efficacy of IBT. Waitlisted opioid-dependent adults were randomly assigned to an IBT (n=25) or Waitlist Control (WLC; n=25) condition, each 12 weeks in duration. IBT participants received BUP maintenance with bi-monthly visits to the clinic for staff-observed dose ingestion and the remaining doses dispensed at home via Med-O-Wheel. They also received IVR-generated nightly check-in calls as well as random call-backs. WLC participants remained on the waitlist of their local clinic. All participants completed 4-, 8- and 12-week follow-up assessments. Our primary outcome was the percent of participants in each group providing urine specimens that tested negative for illicit opioids at these monthly assessments.

Participants randomized to receive IBT provided significantly more illicit opioid-negative urine specimens than those randomized to WLC at Weeks 4 (88% vs. 0%), 8 (84% vs. 0%), and 12 (68% vs. 0%) assessments (p's<.001). They also demonstrated greater reductions in past-month frequency of IV use (p<0.001). We also saw evidence of unexpected IBT-related benefits beyond abstinence, particularly in the area of psychological distress. IBT participants demonstrated greater reductions on the Beck Depression Inventory (BDI), with IBT participants reporting lower levels of depression at Weeks 4, 8, and 12 (p's≤.01). A similar pattern was seen on the Addiction Severity Index (ASI) Psychiatric subscale (p=.02). These data suggest that IBT may produce improvements in psychological symptom severity among waitlisted opioid-dependent individuals, even without formal counseling.

Study retention was high among IBT and control participants (92% and 80%, respectively) and did not differ between groups (p=.42). IBT participants' adherence with the treatment components was also excellent. For example, participants took 99% of scheduled doses and completed 96% of daily calls, which averaged 1.24±0.4 minutes. They satisfied 96% of random call-backs, with 85% of specimens collected at those random visits negative for illicit opioids. The HIV+HCV application was associated with improvements in HIV- and HCV-related knowledge, and these improvements also persisted though 12-week follow-up. Finally, IBT participants' ratings of treatment satisfaction were high (4.6±0.7 on a 5-point scale).

The primary outcomes from this pilot study were published on December 22, 2016 in *The New England Journal of Medicine* (Sigmon et al., 2016), which was followed by a published letter in *NEJM* on March 9 (Sigmon et al., 2017). Our data were also presented in the NIDA Deputy Director's recent congressional testimony to the House Committee on Energy and Commerce on March 21, 2017 (https://www.drugabuse.gov/about-nida/legislative-activities/testimony-to-congress/2017/research-use-misuse-fentanyl-other-synthetic-opioids), as well as NIDA Director Dr. Volkow's annual report to NIH Council on February 10 (https://www.drugabuse.gov/sites/default/files/ volkowfeb2016council talk.pdf). There has also been extensive coverage in the lay media, including an article on the study by the Associated Press' chief medical writer Marilynn Marchione(https://www.usnews.com/news/news/articles/2016-12-21/a-novel-approach-fighting-painkiller-addiction-at-home). These highly promising pilot data suggest that providing technology-supported IBT to waitlisted opioid-dependent individuals may reduce individual and societal risks during delays to comprehensive treatment, representing substantial potential to produce a large public health

impact. Importantly, however, our initial study involved an extremely limited 12-week treatment duration. Many individuals experience much longer waitlist delays. For example, in our state's first and still largest methadone service, for which Dr. Sigmon is Director, we have increased our capacity from 400 to 1,000 patients in the past several years and yet our clinic still has a waitlist of 6-8 months. Thus, it is essential to evaluate IBT over the durations likely to be encountered in the real world. The proposed RCT will provide critical information on IBT efficacy over the durations of treatment delays that exist in the real world.

References*. Include references to prior human or animal research and references that are relevant to the design and conduct of the study.*

Abrahamsson, T., Widinghoff, C., Lilliebladh, A., Gedeon, C., Nilvall, K., Hakansson, A. (2015). Interim buprenorphine treatment in opiate dependence: A pilot effectiveness study. *Subst Abus.* [Epub ahead of print].

Dunn, K.E., Saulsgiver, K.A., Patrick, M.E., Heil, S.H., Higgins, S.T., & Sigmon, S.C. (2013). Characterizing and improving HIV and hepatitis knowledge among primary prescription opioid abusers. *Drug Alcohol Depend, 133*(2), 625-632.

Fiellin, D.A., Pantalon, M.V., Chawarski, M.C., Moore, B.A., Sullivan, L.E., et al. (2006). Buprenorphine maintenance in primary care: A randomized controlled trial of counseling conditions and medication dispensing. *NEJM, 355*(4), 365-374.

Helzer, J.E., Rose, G.L., Badger, G.J., Searles, J.S.,Thomas, C.S., et al. (2008). Using Interactive Voice Response to Enhance Brief Alcohol Intervention in Primary Care Settings. *J Stud Alcohol Drugs, 69,* 251-258.

Higgins, S.T., Stitzer, M.L., Bigelow, G.E., & Liebson, I.A. (1986). Contingent methadone delivery: Effects on illicit opiate use. *Drug Alc Depend, 17*, 311-322.

Jasinski, D.R., Pevnick, J.S., & Griffith, J.D. (1978). Human pharmacology and abuse potential of the analgesic buprenorphine. *Arch Gen Psychiatry, 35*, 501–516.

Johnson, R.E., Chutaupe, M.A., Strain, E.C., Walsh, S.L., Stitzer, M.L. et al. (2000). A comparison of levomethadyl acetate, buprenorphine, and methadone for opioid dependence. *N Engl J Med, 343*(18):1290-1297.

Johnson, R.E., Strain, E.C., & Amass, L. (2003). Buprenorphine: How to use it right. *Drug Alc Depend, 70*, S59-S77.

Krook, A.L., Brørs, O., Dahlberg, J., Grouff, K., Magnus, P., Røysamb, E., & Waal, H. (2002). A placebo-controlled study of high dose buprenorphine in opiate dependents waiting for medication-assisted rehabilitation in Oslo, Norway. *Addiction, 97*(5), 533-542.

McClure, E.A., Acquavita, S.P., Harding, E., & Stitzer, M.L. (2013). Utilization of communication technology by patients enrolled in substance abuse treatment. *Drug Alc Depend, 129*, 145-150.

Moore, B.A., Fazzino, T., Barry, D.T., Fiellin, D.A., Cutter, C.J., et al. (2013). The Recovery Line: A pilot trial of automated, telephone-based treatment for continued drug use in methadone maintenance. *J Subst Abuse Treat, 45*, 63-69.

Rose, G.L., Skelly, J.M., Badger, G.J., Ferraro, T.A., & Helzer, J.E. (2015b). Efficacy of automated telephone continuing care following outpatient therapy for alcohol dependence. *Addict Behav, 41*, 223-231.

Schwartz, R.P., Alexandre, P.K., Kelly, S.M., O'Grady, K.E., Gryczynski, J., & Jaffe, J.H. (2014). Interim versus standard methadone treatment: A benefit-cost analysis. *J Subst Abuse Treat, 46*(3), 306-314.

Schwartz, R.P., Highfield, D.A., Jaffe, J.H., Brady, J.V., Butler, C.B., et al. (2006). A randomized controlled trial of interim methadone maintenance. *Arch Gen Psychiatry, 63,* 102-109.

Schwartz, R.P., Jaffe, J.H., O'Grady, K.E., Das, B., Highfield, D.A., & Wilson, M.E. (2009a). Scaling-up interim methadone maintenance: Treatment for 1,000 heroin-addicted individuals. *J Subst Abuse Treat, 37,* 362-367.

Schwartz, R.P., Kelly, S.M., O'Grady, K.E., Gandhi, D., & Jaffe, J.H. (2012). Randomized trial of standard methadone treatment compared to initiating methadone without counseling: 12-month findings. *Addiction 107*, 943–952.

Schwartz, R.P., Kelly, S.M., O'Grady, K.E., Gandhi, D., & Jaffe, J.H. (2011). Interim methadone treatment compared to standard methadone treatment: 4-month findings. *J Subst Ab Treat*, 41, 21-29.

Sigmon, S.C. (2015b). Interim treatment: Bridging delays to opioid treatment access. *Prev Med, 80*, 32-36.

Sigmon, S.C., Meyer, A.C., Hruska, B., Ochalek, T., Rose, G., Badger, G.J., Brooklyn, J.R., Heil, S.H., Higgins, S.T., Moore, B.A., & Schwartz, R.P. (2015a). Bridging waitlist delays with Interim Buprenorphine Treatment: Initial feasibility. *Addict Behav, 51*, 136-142.

Sigmon, S.C, Ochalek, T.A, Meyer, A.C, Hruska, B., Heil, S.H, Badger, G.J, … Higgins, S. (2016). Interim buprenorphine vs. waiting list for opioid dependence. *NEJM*, *375*(25), 2504–2505. doi:10.1056/NEJMc1610047

Stacy, J.N., Schwartz, S.M., Ershoff, D., & Shreve, M.S. (2009). Incorporating tailored interactive patient solutions using interactive voice response technology to improve statin adherence: Results of a randomized clinical trial in a managed care setting. *Popul Health Manag, 12*(5), 241-254.

Tacke, U., Uosukainen, H., Kananen, M., Kontra, K., & Pentikäinen, H. (2009). A pilot study about the feasibility and cost-effectiveness of electronic compliance monitoring in substitution treatment with buprenorphine–naloxone combination. *J Opioid Manag, 5*, 321–329.

Uosukainen, H., Pentikainen, H., & Tacke, U. (2013). The effect of an electronic medicine dispenser on diversion of buprenorphine-naloxone: Experience from a medium-sized Finnish city. *J Subst Ab Treat, 45,* 143-147.

Volkow, N.D., Frieden, T.R., Hyde, P.S., Cha, S.S., 2014. Medication-assisted therapies – Tackling the opioid-overdose epidemic. *NEJM, 370*, 2063-2066.

Walsh, S.L., Preston, K.L., Bigelow, G.E., & Stitzer, M.L. (1995). Acute administration of buprenorphine in humans: Partial agonist and blockade effects. *J Pharmacol Exp Ther, 274*, 361-372.

Walsh, S.L., Preston, K.L., Stitzer, M.L., Cone, E.J., & Bigelow, G.E. (1994). Clinical pharmacology of buprenorphine: Ceiling effects at high doses. *Clin Pharmacol Ther, 55,* 569-580.

Objectives: *Clearly state the primary and secondary objective(s) of the study.*

Our primary objective is to determine whether IBT effects in reducing illicit opioid use and other risk behaviors endure long enough to constitute meaningful improvement in people's lives while they await entry into traditional treatment.

METHODS AND PROCEDURES

Study Design: *Describe the research design, including a description of any new methodology and its advantage over existing methodologies.*

In this parallel-group randomized controlled trial (RCT), we will evaluate 6-month IBT efficacy in 100 waitlisted opioid-dependent adults. Participants randomly assigned to IBT (n=50) will receive (a) BUP maintenance with bi-monthly visits for observed medication ingestion and the remaining doses dispensed via Med-O-Wheel. (b) Daily monitoring calls via IVR, (c) IVR-generated random call-backs, and (d) HIV+Hepatitis C education via iPad. WLC participants will remain on the waitlist for their treatment of choice. Participants in both groups will complete monthly follow-up assessments. At all scheduled, random and follow-up assessment visits, urine specimens will be collected under staff observation and analyzed immediately for opioid and non-opioid drugs. We hypothesize that IBT participants will achieve significantly greater biochemically-verified illicit opioid abstinence relative to WLC. Secondary outcomes will include IV opioid and other drug use, psychosocial functioning (e.g., ASI subscales reflecting legal, employment, medical, psychiatric, drug and family/social severity), psychological distress (e.g., BDI, BAI, BSI) and several IBT-specific measures (e.g., BUP adherence, IVR utilization, patient satisfaction).

Procedures: *Describe all procedures (sequentially) to which human participants will be subjected. Identify all procedures that are considered experimental and/or procedures performed exclusively for research purposes. Describe the types, frequency and duration of tests, study visits, interviews, questionnaires, etc. Include required screening procedures performed before enrollment and while on study. Please provide in table, list or outline format for ease of review. (describe and attach all instruments)*

Note: A clinical research protocol may involve interventions that are strictly experimental or it may involve some aspect of research (e.g., randomization among standard treatments for collection and analysis of routine clinical data for research purposes). It is important for this section to distinguish between interventions that are experimental and/or carried out for research purposes versus those procedures that are considered standard therapy. In addition, routine procedures performed solely for research purposes (e.g., additional diagnostic/follow-up tests) should be identified.

Assessments. The intake assessment will include a Drug History Questionnaire, ASI (McLellan et al., 1985), psychoactive substance dependence section of the DSM-5 (APA, 2013), Brief Symptom Inventory (Derogatis, 1993), Beck Depression and Anxiety Inventories (Beck et al., 1988, 1996), Michigan Alcoholism Screening Test (Selzer, 1971) and a Time-Line Followback of past-month use of opioids and other drugs (Sobell & Sobell, 1992). Participants will also complete a medical history, receive a brief

physical exam, and provide a urine specimen (see *Biochemical monitoring*). Follow-up assessments will consist of abbreviated versions of the intake, will include urinalysis, and be administered monthly during the 6-month trial. A participant satisfaction questionnaire will also be administered at Week 24. Participants will receive $30 per assessment. This level of compensation has permitted us to achieve high levels (~90%) of follow-up compliance in our IBT and other treatment development studies (Higgins et al., 1995; Sigmon et al., 2016).

<u>Interim Buprenorphine Treatment (IBT, n=50)</u>. Participants randomly assigned to the 24-week IBT condition will receive the following treatment components:

Buprenorphine via computerized dispensing. IBT participants will receive buprenorphine/naloxone sublingual tablets (Amneal Pharmaceuticals, Bridgewater, NJ), which will be obtained through our university hospital's investigational pharmacy. Induction will occur in Week 1, with individualized dose adjustments at daily visits determined using the Clinical Institute Narcotic Assessment (Peachey & Lei, 1988) with the aim of stabilizing participants on a dose sufficient to achieve withdrawal suppression without intoxication or sedation. We have successfully used these procedures in our prior BUP studies (Sigmon et al., 2009, 2013, 2015c, 2016). At each visit, the research nurse will assess withdrawal and agonist effects and the RA will collect urine and breath samples. During stabilization, the RA will also instruct IBT participants on how to use the IVR system and Med-O-Wheel and have them complete HIV+Hepatitis Education (described below). Following stabilization, participants will visit the clinic every two weeks (i.e., Weeks 2, 4, 6, etc.) to provide a urine specimen, ingest that day's dose under nurse observation, and complete measures of opioid and other drug use via Time-Line Followback. They will receive the doses for the upcoming 13-day interval in the Med-O-Wheel (Addoz, Forssa, Finland) for ingestion at home. Participants will be instructed to bring the device with them to each study visit and reminded throughout the study that nonadherence may be grounds for discharge. Participants can also return to the clinic between scheduled visits should any concerns arise or a dose evaluation be needed.

IVR daily monitoring. The IVR system will contact participants each evening at a convenient time identified by them to assess any opioid, other drug or alcohol use, as well as opioid craving and withdrawal. Instances of use, craving or withdrawal will prompt additional detailed questions (e.g., type and amount used, severity of craving and withdrawal), as well as provision of information about support meetings taking place in the community. Participants can make inbound calls to the system at any time to complete their daily check-in if they anticipate missing the call or if it is more convenient. They can also reach study staff at any time on our on-call mobile phone.

Random call-backs via IVR. Participants will be contacted by the IVR system approximately once per two-week dosing interval on a random basis and instructed to return to the clinic (typically within 12 hrs but this can be individualized as needed). For each random call-back, they will refrain from taking that day's dose and instead bring in their Med-O-Wheel to the clinic and ingest their medication under nurse observation. The research nurse will conduct a pill count and inspect the device for any evidence of tampering or nonadherence, and participants will provide breath and urine specimens (see *Biochemical monitoring*).

HIV+Hepatitis education. Using an interactive iPad application developed by us, during stabilization week participants will complete a baseline assessment of HIV and hepatitis knowledge that includes a modified version of the HIV/AIDS Knowledge Test (Marsch et al., 2005), a Hepatitis C knowledge test developed by our group, and VAS items assessing perceived risk, disease transmission and risk behaviors (Dunn et al., 2013). Upon completion of this Pre-Test, the application will provide immediate corrective feedback and explanations for any incorrect items (excluding VAS items). Participants will then view an interactive flipbook ("HIV/AIDS Basics", Aids.gov) and a brief video ("What is Hepatitis C and how is it diagnosed?", Foundation for AIDS Research), also delivered by iPad. They will complete a Post-Test assessment immediately following this educational module, and staff will offer condoms and information on free HIV and hepatitis testing in the community. The HIV+HCV knowledge Post-Test will be repeated at Weeks 12 and 24.

Biochemical monitoring. At each visit, urine specimens will be collected under observation of same-sex staff and immediately analyzed for opioids (BUP, methadone, oxycodone, hydrocodone, hydromorphone, heroin) and other drugs (cocaine, amphetamines, benzodiazepines, cannabinoids) via enzyme multiplied immunoassay (Microgenics, Fremont, CA). Participants will also provide a breath sample to assess recent alcohol use (ALCO-SENSOR III, Intoximeters, Inc., St. Louis, MO).

<u>Waitlist Control (WLC, n=50)</u>. Our aim in the present proposal is to utilize a WLC condition that generally reflects the waitlist conditions received by individuals in the real world. Thus, participants

randomized to the WLC condition will remain on the waitlist for their treatment of choice but complete the same monthly follow-up assessments with urinalysis, as described above for IBT participants. Participants will receive $30 per assessment.

For research involving survey, questionnaires, etc.: *Describe the setting and the mode of administering the instrument and the provisions for maintaining privacy and confidentiality. Include the duration, intervals of administration, and overall length of participation. (describe and attach all instruments)*

☐ ***Not applicable***

Study procedures will be conducted at UVM Buprenorphine Research Clinic, which has been the site of BUP research for the past 25 years. The clinic is located in our University Medical Center's outpatient building and is contiguous with our other research clinics for cocaine dependence and smoking cessation as well as our methadone clinic for which Dr. Sigmon is Director. IBT participants will visit the clinic every 2 weeks while receiving the IBT package described above. WLC participants will remain on the waitlist for their treatment of choice, though they will complete the same scheduled follow-up assessments as IBT participants. WLC participants who have not entered treatment by Week 24 will be offered the opportunity to cross over to IBT at that time, contributing additional within-subject data with which to evaluate the efficacy of the IBT intervention. The intake assessment will include a Drug History Questionnaire, ASI (McLellan et al., 1985), psychoactive substance dependence section of the DSM-5 (APA, 2013), Brief Symptom Inventory (Derogatis, 1993), Beck Depression and Anxiety Inventories (Beck et al., 1988, 1996), Michigan Alcoholism Screening Test (Selzer, 1971) and a Time-Line Followback of past-month use of opioids and other drugs (Sobell & Sobell, 1992). Participants will also complete a medical history, receive a brief physical exam, and provide a urine specimen (see *Biochemical monitoring*). Follow-up assessments will consist of abbreviated versions of the intake, will include urinalysis, and be administered monthly during the 6-month trial (i.e., at Weeks 4, 8, 12, 16, 20, and 24).

Statistical Considerations: *Delineate the precise outcomes to be measured and analyzed. Describe how these results will be measured and statistically analyzed. Delineate methods used to estimate the required number of subjects. Describe power calculations if the study involves comparisons. Perform this analysis on each of the primary and secondary objectives, if possible.*

Statistical methods. IBT and WLC groups will be compared on baseline characteristics using analyses of variance for continuous and chi-square tests for categorical variables. If characteristics differ significantly and are predictive of outcome, they will be considered as covariates in subsequent analyses. The primary outcome will be the percent of participants in IBT and WLC conditions with biochemically-confirmed illicit opioid abstinence at the 24-week assessment, evaluated based on a chi square test. Primary analyses will include all randomized subjects independent of early dropout and with missing urine specimens considered positive for illicit opioids, consistent with an intent-to-treat approach (Armitage, 1983). We hypothesize that participants receiving IBT will achieve significantly greater biochemically-verified illicit opioid abstinence relative to WLC. If applicable, logistic regression will be used to adjust for baseline covariates confounded with treatment group. Though not the primary outcome, abstinence at the other monthly assessments will be compared between groups with Bonferroni-adjusted chi square tests to control experiment-wise type I error rate below $\alpha=.05$. Within the IBT group, generalized estimating equation (SAS, PROC GENMOD) will be used to characterize the temporal trend in abstinence across monthly assessments. Additional outcomes will include IV opioid use, other (non-opioid) drug use, retention, BUP adherence, IVR utilization, and patient satisfaction. Repeated measures analyses of variance (SAS, PROC MIXED) will be used to compare groups on continuous outcomes (e.g., past-month frequency of illicit opioid and IV use, ASI subscales, BDI, HIV+HCV knowledge). If significant treatment by time interactions are detected, partial F-tests will be used to examine simple effects of treatment at each assessment. Additional repeated measures analyses will characterize temporal patterns in IBT-specific outcomes (e.g., BUP adherence, IVR utilization, satisfaction). Statistical analyses will be performed using SAS statistical software Version 9.4.

Sample size justification. The proposed sample size is based on having sufficient power for detecting treatment differences for both primary and secondary outcomes. Regarding the primary outcome measure, the percent of participants negative for illicit opioids at the 24-week assessment, power is estimated to be greater than 95% using $\alpha=.05$ if true abstinence rates are as low as 60% vs. 15% for IBT and WLC groups, respectively. These estimates are more conservative than our R34 outcomes, with lower abstinence predicted in IBT as we increase the duration to 24 weeks. Furthermore, power is estimated to be 80% or more to detect group differences as low as 30% in 24-week abstinence rates (ES=0.56). For secondary outcomes, effect sizes range from small to moderate. The estimated mean

differences and minimum detectable effect sizes corresponding to 80% power (α=.05) for detecting treatment group differences in the change from baseline to Week 24 for representative ASI subscales are: Employment=.118 (ES=0.32), Psych=.107 (ES=0.51), Legal=.062 (ES=0.45), and Drug=.079 (ES=0.72). Similarly, power is 80% for detecting a mean difference of 5.5 units in the 24-week change in BDI (ES=0.42), which is smaller than we observed in our pilot trial. Lastly, power is estimated to be 80% to detect a mean absolute difference of 8.5% in the pre-post change in HIV knowledge scores (ES=0.54). It should be noted that these effect sizes (Cohen d's) are expressed relative to the between-subject variability at baseline [i.e. $(\Delta_{IBT}-\Delta_{WLC})/\sigma_{between\text{-}subject}$], not the variability in the change scores [i.e. $(\Delta_{IBT}-\Delta_{WLC})/\sigma_{\Delta}$](Becker, 1988; Morris, 2008). The actual computation accounts for the correlation observed across repeated assessments for each outcome observed in our pilot data which is responsible for the different estimates of detectable ES's.

Risks/Benefits: *Describe any potential or known risks. This includes physical, psychological, social, legal or other risks. Estimate the probability that given risk may occur, its severity and potential reversibility. If the study involves a placebo or washout period, the risks related to these must be addressed in both the protocol and consent. Describe the planned procedures for protecting against or minimizing potential risks and assess their likely effectiveness. Where appropriate, discuss plans for ensuring necessary medical or professional intervention in the event of adverse effects to the subjects. Discuss the potential benefits of the research to the subjects and others. Discuss why the risks to the subjects are reasonable in relation to the anticipated benefits to subjects and others. Discuss the importance of the knowledge gained or to be gained as a result of the proposed research and why the risks are reasonable in relation to the knowledge that reasonably may result. If there are no benefits state so.*

Risks include breach of confidentiality and any side effects associated with the study medication (i.e., buprenorphine). Breach of confidentiality: Study data include medical and psychiatric histories and biological measures of alcohol and illicit drug use and pregnancy. The likelihood of a breach of confidentiality is low as we will take precautions to minimize this risk as described below under Adequacy of Protection against Risk.

Side effects of buprenorphine: The side effects of buprenorphine include light-headedness, dizziness, sedation, lethargy, changes in sexual ability, nausea, vomiting, sweating, euphoria, constipation, respiratory depression, flushing of the face, skin itchiness or redness, darkening of the skin and/or swelling, bradycardia, headache, yawning, tearing, runny nose, muscle tremor, dilated or constricted pupil, restlessness, diarrhea, hypertension, hypotension, or potentially elevated liver enzyme levels (particularly among subjects with a history of hepatitis). The administration of the partial opioid agonist, buprenorphine, in individuals physically dependent on opioids should not result in acute toxicity because these individuals are tolerant to such drug effects. There also is a ceiling on the agonist effects of partial agonists; thus, the agonist effects of the partial agonist, buprenorphine, are considered to be safer than full agonists. Because buprenorphine is a partial agonist, it could also function as an antagonist and promote withdrawal symptomatology. We will administer buprenorphine in accordance with standard practice (see Methods) and, based on our previous experience in treating opioid-dependent individuals with this medication (Sigmon et al., 2009; Sigmon et al., 2013, 2015, 2016), we do not anticipate that buprenorphine-precipitated withdrawal or sedation will pose a problem.

Protection Against Risk: (1) To protect confidentiality, the guidelines stated in Title 42 of the Code of Federal Regulations, Part 2, “Confidentiality of Alcohol and Drug Abuse Records” will be followed. As stated in these regulations, subjects will be given a notice of federal confidentiality requirements (which will be included in the consent form). All records will be locked in file cabinets kept on site behind locked doors. Except for intake material, subjects' names (i.e., first and last names) will not be attached to the data forms. A central code/data base linking subject number with subject names will be kept, which will be available only to specified staff.

(2) In order to protect participants from any adverse effects of buprenorphine, a number of safeguards will be in place. First and most generally, subjects will be screened thoroughly at intake using medical, psychiatric, drug abuse, and cognitive interviews and self-reports. They will have a complete physical exam, and follow-up interviews and tests may be ordered to clarify results. The results of all tests will be reviewed by the medical director (Dr. Brooklyn) and the PI (Dr. Sigmon). Thus, we will document that the patient is healthy to participate in the proposed study. To prevent subjects who may have an insufficient level of opioid dependence from participating in these studies, they will have to meet several criteria (e.g., DSM-5 criteria for opioid dependence and FDA qualification criteria for buprenorphine treatment, including a history of opioid dependence and significant current opioid use). All nursing and research staff will be trained by medical staff in detecting adverse effects. If a subject has any untoward effects, the Study Physician and PI will be contacted. Study Physician Dr. Brooklyn will be on-call continuously for advice and assistance in the event that adverse effects occur. Dr. Brooklyn has been working with our previous

buprenorphine projects over the last 15 years, is a buprenorphine provider, is Medical Director of the Chittenden Clinic methadone program, and he has extensive experience with the clinical use of buprenorphine as a Vermont buprenorphine provider and trainer. The emergency room for the University of Vermont Medical Center is located approximately one block away from the UHC.

(3) Finally, patients are free not to participate in this study or to withdraw from it at any time. If they decide not to participate in the study, we will be glad to discuss with them other treatments that may be available in Vermont, including residential, outpatient, and medication-assisted treatment options. If patients decide not to participate in this study or to withdraw from this study, their decision will not prejudice their future medical care at the University of Vermont or University of Vermont Medical Center. The investigators also retain the right to terminate patients' participation in the study if in their judgment continued participation would put them in physical or psychological danger. Additional details on our data and safety monitoring of the proposed research to ensure the safety of subjects is provided in the below section entitled "Data and Safety Monitoring Plan".

Potential Benefits to Participants and Others

Volunteers may benefit by initiating abstinence from illicit opioids during their study participation, including experiencing a reduction in the wide range of medical, financial, psychosocial, and legal consequences associated with illicit opioid abuse. By improving treatment access for opioid abuse and dependence, the proposed research also stands to benefit public health in general by reducing the vast economic and societal costs associated with opioid abuse (e.g., health service utilization and costs, criminal activity, contraction of preventable diseases such as HIV and hepatitis). In addition, there are potential scientific benefits to be gained by expanding our empirical knowledge on how to mitigate gaps in treatment access among opioid-dependent individuals. Overall, the individual participant, the medical and scientific communities, and society in general may benefit by our efforts to develop an interim buprenorphine treatment for patients awaiting agonist maintenance. As such, the risks to which individuals may be exposed as a function of their research participation are reasonable in relation to the anticipated benefits.

Importance of the Knowledge to be Gained

The proposed project has the potential to contribute a novel and effective technology-assisted pharmacotherapy protocol that can be widely disseminated to bridge gaps in access to life-saving opioid treatment. Thus, knowledge gained from this research may significantly enhance the accessibility, implementation and effectiveness of drug abuse treatment more generally. Consequently, the risk/benefit ratio is favorable. The risks to which individuals are exposed as a consequence of their research participation are generally less than that associated with continuing their ongoing abuse of illicit opioids. In contrast, the potential and probable benefits to be derived by society in general and by opioid abusers as a group are considerable. In summary, conducting this research seems well justified.

Therapeutic Alternatives: *List the therapeutic alternatives that are reasonably available that may be of benefit to the potential subject and include in the consent form as well.*

☐ ***Not Applicable***

Participants are free not to participate in this study or to withdraw from it at any time. If they decide not to participate in the study, we will be glad to discuss with them other treatments that may be available in Vermont, including residential, outpatient, and medication-assisted treatment options.

Data Safety and Monitoring: *The specific design of a Data and Safety Monitoring Plan (DSMP) for a protocol may vary extensively depending on the potential risks, size, and complexity of the research study. For a minimal risk study, a DSMP could be as simple as a description of the Principal Investigator's plan for monitoring the data and performance of safety reviews or it could be as complex as the initiation of an external, independent Data Safety and Monitoring Board (DSMB). The UVM/UVM Medical Center process for review of adverse events should be included in the DSMP.*

Patient eligibility and status. All intake data collection will be conducted by a trained bachelor's-level Research Assistant (RA) using specialized forms and procedures. Medical screening data will be reviewed by the Study Physician. All intake information will be reviewed by the PI, who will determine participant eligibility. Only trained and IRB-approved research staff will complete informed consent with eligible and willing participants. The status of all active participants will be reviewed at weekly meetings between the PI, Co-investigators and RAs.

Rigorous data management/Quality assurance. The majority of study data collection will be conducted using self-report questionnaires. Randomly selected data will be checked by the RAs for completeness and to ensure quality (i.e., no appearance of rote answers, etc.). In terms of standard operating procedures at the clinic, all assessments will be administered by trained research staff. All subject data will be maintained in secure filing cabinets behind locked doors in order to protect confidential subject information. Safe places will include locked filing cabinets or locked rooms that will be accessible

only to study personnel. Full subject names will not be listed on the outside of the binders in order to protect the identity of study participants. Moreover, all data that are entered into spreadsheets and databases, in preparation for data analyses, will be entered twice. That is, two separate individuals will enter the data into databases, and a comparison between data entries will be conducted to detect data entry errors. All discrepancies in data entry will be checked against the raw data source, and the correct data entry will be used. All data that are entered into spreadsheets and databases will be coded by subject ID number and not by subject name. Additionally, all entered data will be backed up on an external hard drive or a secure project server at least weekly. The biostatistician and PI will discuss any problems at monthly data meetings. Additional meetings will be conducted on an as-needed basis. An original copy of the data will be retained at the clinic should anything happen to the document during transmission.

Auditing procedures. Review of any problems related to quality of data collection, transmission or analyses and of any AEs and SAEs that occurred during the past week will occur at weekly research staff meetings. Interim analyses of efficacy data will be conducted when half the subjects have been entered or at other times based on the joint discretion of the PI, Co-I and biostatistician.

Adverse Event and Unanticipated Problem (UAP) Reporting: *Describe how events and UAPs will be evaluated and reported to the IRB. All protocols should specify that, in the absence of more stringent reporting requirements, the guidelines established in the Committees on Human Research "Adverse Event and Unanticipated Problems Reporting Policy" will be followed. The UVM/UVM Medical Center process for review of adverse events and UAPs to subjects or others should be included in the DSMP.*

AEs and SAEs will be assessed at each clinic visit by a trained RA or nurse. Any SAE will be brought to the attention of the PI as soon as possible and not longer than 24 hrs. Any SAE, whether or not related to study intervention, will be reported to the IRB using the UVM Adverse Event Reporting Document within 5 days of the event. The IRB will make a determination as to whether additional reporting requirements are needed. Any SAEs will also be summarized in the yearly IRB continuing review, including a review of frequency and severity.

Withdrawal Procedures: *Define the precise criteria for withdrawing subjects from the study. Include a description of study requirements for when a subject withdraws him or herself from the study (if applicable).*

Completing study visits and providing urine specimens as scheduled are necessary in order for subjects to receive the study medication. Thus, if the subject fails to attend scheduled visits, or does not submit regularly scheduled urine specimens, or if there is any evidence of nonadherence to the medication regimen, they will be withdrawn from the study. If they are discharged from the study, subjects will be expected to return all remaining medication and the Med-O-Wheel device to study staff immediately. Females will be tested for pregnancy prior to and during the study. Should a subject become pregnant during the trial, her participation will be terminated and she will be assisted with accessing treatment at the medical center's high-risk pregnancy clinic.

Patients are free not to participate in this study or to withdraw from it at any time. If they decide not to participate in the study, we will be glad to discuss with them other treatments that may be available in Vermont, including residential, outpatient, and medication-assisted treatment options. If patients decide not to participate or to withdraw from this study, their decision will not prejudice their future medical care at UVM or UVM Medical Center.

Sources of Materials: *Identify sources of research material obtained from individually identifiable human subjects in the form of specimens, records or data. Indicate whether the material or data will be obtained specifically for research purposes or whether use will be made of existing specimens, records or data.*

Research materials will include questionnaires, structured clinical interviews, expired air samples for analyzing breath alcohol levels, urine samples for analyzing recent drug use and pregnancy status. All data will be collected for research purposes only. All data collection will be conducted by a trained bachelor's-level Research Assistant (RA) with special training on all forms and procedures. All information will be reviewed by the PI, who will determine participant eligibility and complete informed consent with eligible and willing participants. Subject data will be maintained in secure filing cabinets behind locked doors in order to protect confidential subject information. Safe places will include locked filing cabinets or locked rooms that will be accessible only to study personnel. Full subject names will not be listed on the outside of the binders in order to protect the identity of study participants. Subject data and subject identifiers will only be accessible to approved research staff.

DRUG AND DEVICE INFORMATION

Investigators are encouraged to consult the UVM Medical Center Investigational Pharmacy Drug Service (847-4863) prior to finalizing study drug/substance procedures.

Drug (s) ☐ **Not applicable**

Drug name – generic followed by brand name and common abbreviations. Availability – Source and pharmacology; vial or product sizes and supplier. If a placebo will be used, identify its contents and source. (attach investigational drug brochure)

IBT participants will receive buprenorphine/naloxone (Suboxone/Naloxone; bup) sublingual tablets (Amneal Pharmaceuticals, Bridgewater, NJ), which will be obtained through the UVM Medical Center's Investigational Pharmacy. Buprenorphine is an FDA approved and widely used medication for treating opioid use disorder.

Preparation: Reconstitution instructions; preparation of a sterile product, compounded dosage form; mixing guidelines, including fluid and volume required. Identify who will prepare.

UVM Medical Center Outpatient Pharmacy at University Health Center

Storage and stability – for both intact and mixed products.

Administration – Describe acceptable routes and methods of administration and any associated risks of administration.

Daily sublingual administration

Toxicity – Accurate but concise listings of major toxicities. Rare toxicities, which may be severe, should be included by indicated incidence. Also adverse interactions with other drugs used in the protocol regimen as well as specific foods should be noted. Address significant drug or drug/food interactions in the consent form as well. List all with above details.

No major toxicities

Is it FDA approved: (include FDA IND Number)

1. *in the dosage form specified? If no, provide justification for proposed use and source of the study drug in that form.*

   Y

2. *for the route of administration specified? If no, provide justification for route and describe the method to accomplish.*

   Y

3. *for the intended action?*

   Y

Device (s) x **Not applicable**

Device name and indications (attach investigational device brochure)

Is it FDA approved: (include FDA IDE Number)

1. *for indication specified? If no, provide justification for proposed use and source of the device.*

Risk assessment (non-significant/significant risk) - PI or sponsor needs to assess risk of a device based upon the use of the device with human subjects in a research environment.

SUBJECT CHARACTERISTICS, IDENTIFICATION AND RECRUITMENT

Subject Selection: *Provide rationale for subject selection in terms of the scientific objectives and proposed study design.*

Subjects will be adults with current opioid use disorder who are waitlisted for methadone or buprenorphine treatment

Vulnerable Populations: *Explain the rationale for involvement of special classes of subjects, if any. Discuss what procedures or practices will be used in the protocol to minimize their susceptibility to undue influences and unnecessary risk (physical, psychological, etc.).*

x ***Not applicable***

Number of Subjects: *What is the anticipated number of subjects to be enrolled at UVM/UVM Medical Center and in the case of a multi-center study, with UVM/UVM Medical Center as the lead, the total number of subjects for the entire study.*

The anticipated number of subjects to be enrolled in the study is 100 (50 per experimental condition).

Inclusion/Exclusion Criteria: *Eligibility and ineligibility criteria should be specific. Describe how eligibility will be determined and by whom. Changes to the eligibility criteria at a later phase of the research have the potential to invalidate the research.*

Participants must be ≥18 years old, in good health, meet DSM-5 criteria for opioid use disorder and provide an opioid-positive urine at study intake. Consistent with our prior BUP studies, those with a significant psychiatric or medical illness that may interfere with consent or participation will be excluded, as will those who are pregnant or nursing. Those dependent on sedative-hypnotics will be excluded, due to the medical risks and notably low success rates with sedative-dependent opioid abusers (Stitzer & Chutuape, 1999). Intake information will be reviewed by the PI (Dr. Sigmon) and study physician (Dr. Brooklyn), who will determine and document whether the participant is eligible and healthy to participate.

Inclusion of Minorities and Women: *Describe efforts to include minorities and women. If either minorities or women are excluded, include a justification for the exclusion.*

The study will include minorities and women. We will do all that we can to assure that women and minorities are represented in the research, including using a wide range of recruitment sources to ensure that information regarding this project reaches as diverse of a population as possible (e.g., flyers posted in multiple local neighborhoods, advertisements in the widely-distributed primary local newspaper as well as alternative local papers).

Inclusion of Children: *Describe efforts to include children. Inclusion is required unless a clear and compelling rationale shows that inclusion is inappropriate with respect to the health of the subjects or that inclusion is inappropriate for the purpose of the study. If children are included, the description of the plan should include a rationale for selecting or excluding a specific age range of children. When included, the plan must also describe the expertise of the investigative team in working with children, the appropriateness of the available facilities to accommodate children, and the inclusion of a sufficient number of children to contribute to a meaningful analysis relative to the purpose of the study.* ***If children are excluded*** *then provide appropriate justification. Provide target accrual for this population.*

Children will not be eligible for this study.

For protocols including the use of an investigational drug, indicate whether women of childbearing potential have been included and, if not, include appropriate justification.

N/A

If HIV testing is included specifically for research purposes explain how the test results will be protected against unauthorized disclosure. Include if the subjects are to be informed of the test results. If yes, include the process and provision for counseling. If no, a rationale for not informing the subjects should be included.

x	***Not applicable***

Recruitment: *Describe plans for identifying and recruitment of subjects. All recruitment materials (flyers, ads, letters, etc) need to be IRB approved prior to use.*

The primary referral source will be distribution of an IRB-approved flyer to all CC waitlist individuals informing them about the study. Should this recruitment method ever become insufficient, we can also circulate ads throughout the larger community in order to reach additional patients on wait lists for BUP maintenance via OBOT. Additional sources will include self-referrals, drug abuse clinics, the Vermont State Alcohol and Drug Abuse Office, physicians, local mental health centers, a toll-free number, public service announcements, advertisements in local and alternative newspapers and flyers placed throughout the community. We have successfully recruited participants using these sources in prior studies (Dunn et al., 2008, 2010; Sigmon et al., 2009, 2013, 2015, 2016) and anticipate no difficulties gaining ready access to the sample needed.

Contact between participants and study staff will be initiated by the participants. Potential participants will respond to mailings or advertisements that contain a study description and the name and phone number of the Research Assistant. When potential participants call the Research Assistant, s/he will briefly describe the study and use a brief phone screen to make a preliminary determination about the potential participant's eligibility. Those who are interested in participating and appear to be eligible will be schedule for a longer intake screening that will begin with a full study description of study procedures. Those interested in undergoing study screening will then be provided with a copy of the consent form to read as we go over it with them. Risks and benefits of the study will be described. Potential participants will be asked to paraphrase the consent form and will be asked questions to determine their understanding of key elements of the informed consent. Potential participants who wish to proceed with the interview will be asked to sign the interview consent form and will be given a signed copy of his/her signed consent form.

FINANCIAL CONSIDERATIONS

Expense to Subject: *If the investigation involves the possibility of added expense to the subject (longer hospitalization, extra studies, etc.) indicate in detail how this will be handled. In cases where the FDA has authorized the drug or device company to charge the patient for the experimental drug or device,* ***a copy of the authorization letter from the FDA or sponsor must accompany the application. Final approval will not be granted until the IRB receives this documentation.***
There are very limited circumstances under which study participants may be responsible (either directly or via their insurance) for covering some study-related expenses. If the study participant or their insurer(s) will be billed for any portion of the research study, provide a justification as to why this is appropriate and acceptable. For example, if the study involves treatment that is documented standard of care and not investigational, state so. In these cases, the protocol and the consent should clearly define what is standard of care and what is research.

There are no expenses to the subject for any aspect of the study.

Payment for participation: *Describe all plans to pay subjects, either in cash, a gift or gift certificate. Please note that all payments must be prorated throughout the life of the study. The IRB will not approve a study where there is only a lump sum payment at the end of the study because this can be considered coercive. The amount of payment must be justified. Clarify if subjects will be reimbursed for travel or other expenses.*

	Not applicable

Participants will receive a $30 gift certificate for completing each assessment (at intake and 4, 8, 12, 16, 20, and 24-week follow-up assessments).

***Collaborating Sites**. When research involving human subjects will take place at collaborating sites or other performance sites when UVM/UVM Medical Center is the lead site, the principal investigator must provide in this section a list of the collaborating sites and their Federalwide Assurance numbers when applicable. (agreements may be necessary)*

x	***Not applicable***

INFORMED CONSENT

***Consent Procedures**: Describe the consent procedures to be followed, including the circumstances under which consent will be obtained, who will seek it, and the methods of documenting consent. Specify the form(s) that will be used e.g. consent (if multiple forms explain and place identifier on each form), assent form and/or HIPAA authorization (if PHI is included). These form(s) must accompany the protocol as an appendix or attachment.*
Note: Only those individuals authorized to solicit consent may sign the consent form confirming that the prospective subject was provided the necessary information and that any questions asked were answered.

Only the site-PI (Dr. Sigmon) or trained, IRB-approved research staff will obtain informed consents for these studies. Informed consent will be initiated in a private office in the UHC building. Participants will be given unlimited time to decide if they wish to participate. The site-PI (Dr. Sigmon) and/or trained, IRB-approved research staff will be present and the PI will be continuously available to answer questions and explain all study procedures at any time.

***Information Withheld From Subjects:** Will any information about the research purpose and design be withheld from potential or participating subjects? If so, explain and justify the non-disclosure and describe plans for post-study debriefing.*

x	***Not applicable***

Summary of important changes after Trial 1 commencement:

1.) Given changes in treatment availability in Vermont between the pilot and present studies, the inclusion criterion that participants be waitlisted for agonist maintenance was modified to include participants not currently receiving opioid agonist treatment.

2.) The target sample size was changed to 50 in concert with similar changes requested by funding agency for the parallel Trial 2.

3.) The Brief Pain Inventory (Cleeland, 1989, 1990; Keller et al., 2004) was added to the assessment battery.

4.) The opioid overdose educational module (below) was added to the intervention.

5.) HIV, HCV, and opioid overdose knowledge post-tests were added to the Week 4 assessment.

6.) Compensation for intake and monthly assessments was increased from $30 to $75 to facilitate timely recruitment.

7.) With the onset of the COVID-19 pandemic, the following modifications were made to the study protocol in situations where participants were at high-risk and/or currently experiencing COVID-19 symptoms: participants were permitted meet with study staff less frequently or remotely via phone or UVM's institutionally-supported Zoom platform to reduce the risk of COVID-19 transmission; biochemical samples may be omitted or collected using modified procedures to reduce risk of COVID transmission.

The University of Vermont Committees on Human Research

For Committee Use Only
PROTOCOL NUMBER

Human Subjects Research Protocol

The Common Human Subjects Protocol Cover Form **must** be completed and **attached** to the front of this form. This Protocol form should be completed for any human subjects research proposal that does not have a specific "protocol," such as a grant application. This form must be submitted along with a copy of the complete grant proposal and all the information in this form **must** be consistent with that proposal. This protocol form, once IRB approved, will be the working protocol for that research. **When completing this document, do not refer to page numbers within your grant**. If revisions are necessary during the course of the research, amendments should refer to this protocol form, not the grant proposal. Enter responses for all sections. Check N/A if the section does not apply.

PROTOCOL SUMMARY

Project Title:	Protocol Version Date:
Interim Buprenorphine Treatment to bridge waitlist delays: Stage II evaluation (Trial 2)	07/25/2017

Principal Investigator: Stacey C. Sigmon

Grant Sponsor: National Institute on Drug Abuse   Grant Number: 1R01DA042790 - 01
(For grants routed through UVM, indicate the OSP Proposal ID # located at the top of the OSP Routing Form)

PURPOSE AND OBJECTIVES

Purpose: *The importance of the research and the potential knowledge to be gained should be explained in detail. Give background information.*

Opioid abuse and dependence have reached epidemic proportions in the US, resulting in premature death, criminal activity, and economic costs of over $56 billion annually (Becker et al., 2008; Birnbaum et al., 2011; Clausen et al., 2009; Jones et al., 2013; Shah et al., 2008; SAMHSA, 2010; Wisniewski et al., 2008). The recent increases in opioid-related overdoses are particularly concerning, with 3- and 6-fold increases in overdose deaths related to prescription opioids and heroin, respectively, between 2001 and 2014 (CDC, 2015). Untreated opioid dependence has been associated with unprecedented outbreaks of HIV and Hepatitis C (Strathdee & Beyrer, 2015), and overdose deaths have now surpassed motor vehicle fatalities to become the country's leading cause of injury death (CDC, 2016).

Treatment with agonist medications (e.g., methadone, buprenorphine) represents the most efficacious treatment for opioid dependence and dramatically reduces morbidity, mortality and spread of infectious disease (Schwartz et al., 2013; Stotts et al., 2009). However, demand for treatment far exceeds available capacity in many areas of the country (Friedmann et al., 2003; Harlow et al., 2013; Sigmon, 2014), with ***96% of states in the US having opioid abuse rates that exceed their medication-assisted treatment capacity*** (Jones et al., 2015). The issue of insufficient treatment availability is especially urgent in rural areas (Paulozzi & Xi, 2008; Rintoul et al., 2011; Rosenblum et al., 2011; Sigmon, 2014; Stein et al., 2015). The end result is that opioid-dependent individuals can remain on waitlists for months, during which they are at significant risk for illicit drug use, criminal activity, infectious disease, overdose and mortality (Chun et al., 2008; Clausen et al., 2009; Schwartz et al., 2009b). In one evaluation of survival rates among opioid-dependent individuals who received waitlist vs. immediate entry into methadone treatment, for example, ***opioid treatment waitlists were associated with a 12-fold increase in risk of premature death*** (Peles et al., 2013).

One effort to mitigate individual and societal risks during treatment delays has been to offer interim methadone treatment (IMT; i.e., daily methadone without counseling) to individuals awaiting entry into a methadone program (Sigmon, 2015b). IMT has been shown to produce dramatic reductions in drug use, risk behaviors and criminal activity during treatment delays (Gruber et al., 2008; Schwartz et al., 2006, 2011; Yancovitz et al., 1991). However, few clinics have implemented this approach, likely due to methadone's regulatory and pharmacological limitations. Methadone treatment is limited to licensed specialty clinics and the medication itself has risks of diversion, abuse and overdose with misuse (Luty et

al., 2005). IMT regulations also limit its duration to no more than 120 days, with clinics required to admit patients at that time or discharge them if a treatment slot is still not available. Importantly, they also prohibit take-home doses and require that patients ingest all doses under direct staff observation, resulting in daily clinic visits. These features are incompatible with an environment of constrained resources and have limited the widespread adoption of IMT.

We have developed a novel **Interim Buprenorphine Treatment (IBT)**, aimed at reducing the risks associated with treatment delays while surmounting the limitations noted above for methadone. The intervention includes several key components to permit delivery of life-saving pharmacotherapy while minimizing nonadherence:

(a) The partial opioid agonist buprenorphine (BUP) has a favorable abuse liability and safety profile and it attenuates the effects of other opioids, thereby suppressing illicit use during treatment (Johnson et al., 2003). Importantly, it is also available without the rigid regulations, daily dosing requirements, and 120-day interim-dosing limit required for methadone, making it uniquely compatible with an interim-dosing approach for opioid dependence. Our IBT intervention consists of BUP maintenance with participants' visiting the research clinic bi-monthly to ingest that days dose under nurse observation and then receiving the remaining doses for ingestion at home via a secure computerized portable device (Med-O-Wheel Secure, Addoz, Forssa; Fig 1). The Med-O-Wheel is a portable, tamper resistant pill dispenser. Each day's dose is secured in separate compartments around the cassette and available for a 3-hour window which is programmed by staff. At the specified dosing time, an alarm sounds and the user presses a button on the device cover. The Med-O-Wheel moves the correct dose into place for release. Otherwise the device remains locked until the next specified dosage time and all medication is secured inside the unit. It also includes a visual alarm (i.e., red light on the bottom of the unit) that is activated if there is an attempt to tamper with the device or access to medication outside the preset time window.

(b) An intensive clinical support package is impractical for the resource-constrained settings in which IBT is likely to be delivered. However, one alternative is the rapidly-expanding use of mobile health (*m*Health) platforms, particularly given the increasing number of portable devices with sophisticated functionality (Boyer et al., 2010). *m*Health platforms can extend the reach of health care by permitting delivery of monitoring, education, point-of-care diagnostics and treatment beyond the confines of the medical office. Interactive Voice Response (IVR) systems are particularly promising in that they can provide customized content and support via phone and offer advantages of low cost, consistent delivery, expanded access, 24-hour availability and convenience (Helzer et al., 2008; Rose et al., 2010ab; Stacy et al., 2009). Patients use keypad or voice to respond to prompts and answer questions.

IVR is an ideal choice for IBT. Phone-based systems are compatible with resource-constrained settings as they require no specialized equipment or extensive training. IVR hardware and software can support multiple clinic sites and have no on-site installation costs beyond telephone access. They provide broad access for marginalized populations, as phones are familiar, easy to use, and more widely available than computers. IVR uses an auditory interactive process that is not hampered by low literacy. Privacy is also greater than on a computer screen or written questionnaire. We have developed an IVR system that is compatible with the low-intensity, extended-reach IBT approach. It calls participants nightly to assess any opioid or other drug use, as well as opioid craving and withdrawal. It includes information about self-help meetings in the community and immediate connection with research staff if needed. To maximize participant convenience and adherence, we designed the system to automatically call participants each day rather than requiring them to call into the system; however, it also accepts inbound calls if the participant prefers or anticipates missing the incoming call.

(c) Biochemical verification, typically via urine toxicology, is the most objective method for evaluating recent drug use (Chermack et al., 2000; Fendrich et al., 2004; Kilpatrick et al., 2000; Preston et al., 1997; Wish et al., 1997). Our long-standing protocol involves thrice-weekly urinalysis monitoring during the early months of treatment, followed by a reduction to twice weekly once participants are stable (Higgins, Sigmon et al., 2003). While this rigorous protocol maximizes detection of even low levels of drug use (Cone & Dickerson, 1992), thrice-weekly visits are incompatible with IBT and resource-constrained settings more generally. To balance the rigor of the above procedure with the less-intensive schedule necessary for IBT, we developed a random call-back protocol wherein patients are contacted by IVR at random times and instructed to return to the clinic. Random sampling increases the effectiveness of monitoring, as patients are always in the position of not knowing when the next drug screen will be requested, reducing the possibility that s/he can tailor drug use to subvert monitoring (Harford & Kleber, 1978; Manno, 1986). For each random call-back, the IVR system contacts participants and instructs them to visit the clinic to provide

a staff-observed urine specimen and present their Med-O-Wheel for inspection to ensure there is no evidence of tampering or nonadherence.

(d) Finally, while IBT is intended as a low-intensity, extended-reach paradigm, we believe it is essential to include an intervention to enhance HIV and hepatitis knowledge in this extremely high-risk group. HIV and hepatitis (HCV) are responsible for an estimated 30,000 US deaths annually, and deaths from HCV-related liver complications are increasing (CDC, 2008, 2011; Larney et al., 2012). Brief psychosocial interventions that focus on increasing knowledge about disease prevention are appealing in that they can be done in a single session, do not require extensive counselor training, and have been associated with decreases in self-reported risk behaviors (Copenhaver et al., 2006; Meader et al., 2010).

Our team has developed a single-visit intervention that produces significant improvements in HIV and HCV knowledge in drug-dependent adults (Dunn et al., 2013; Heil et al., 2005; Herrmann et al., 2013). Participants complete a baseline knowledge assessment (Pre-Test) followed by corrective feedback, both administered via iPad. They then complete an interactive HIV flipbook (Fig 2) and animated HCV video, followed by a repeat administration of the knowledge assessment (Post-Test). Finally, to evaluate whether improvements in knowledge persist in the months following the educational intervention, the Post-Test is administered again at 4 and 12 weeks following intake.

Supporting preliminary data. We recently completed a small pilot study evaluating the initial efficacy of IBT. Waitlisted opioid-dependent adults were randomly assigned to an IBT (n=25) or Waitlist Control (WLC; n=25) condition, each 12 weeks in duration. IBT participants received BUP maintenance with bi-monthly visits to the clinic for staff-observed dose ingestion and the remaining doses dispensed at home via Med-O-Wheel. They also received IVR-generated nightly check-in calls as well as random call-backs. WLC participants remained on the waitlist of their local clinic. All participants completed 4-, 8- and 12-week follow-up assessments. Our primary outcome was the percent of participants in each group providing urine specimens that tested negative for illicit opioids at these monthly assessments.

Participants randomized to receive IBT provided significantly more illicit opioid-negative urine specimens than those randomized to WLC at Weeks 4 (88% vs. 0%), 8 (84% vs. 0%), and 12 (68% vs. 0%) assessments (p's<.001). They also demonstrated greater reductions in past-month frequency of IV use (p<0.001). We also saw evidence of unexpected IBT-related benefits beyond abstinence, particularly in the area of psychological distress. IBT participants demonstrated greater reductions on the Beck Depression Inventory (BDI), with IBT participants reporting lower levels of depression at Weeks 4, 8, and 12 (p's$\leq$.01)(Fig 4; dotted line denotes a score suggesting need for treatment; Sprinkle et al., 2002). A similar pattern was seen on the Addiction Severity Index (ASI) Psychiatric subscale (p=.02). These data suggest that IBT may produce improvements in psychological symptom severity among waitlisted opioid-dependent individuals, even without formal counseling.

Study retention was high among IBT and control participants (92% and 80%, respectively) and did not differ between groups (p=.42). IBT participants' adherence with the treatment components was also excellent. For example, participants took 99% of scheduled doses and completed 96% of daily calls, which averaged 1.24$\pm$0.4 minutes. They satisfied 96% of random call-backs, with 85% of specimens collected at those random visits negative for illicit opioids. The HIV+HCV application was associated with improvements in HIV- and HCV-related knowledge, and these improvements also persisted though 12-week follow-up. Finally, IBT participants' ratings of treatment satisfaction were high (4.6$\pm$0.7 on a 5-point scale).

The primary outcomes from this pilot study were published on December 22, 2016 in *The New England Journal of Medicine* (Sigmon et al., 2016), which was followed by a published letter in *NEJM* on March 9 (Sigmon et al., 2017). Our data were also presented in the NIDA Deputy Director's recent congressional testimony to the House Committee on Energy and Commerce on March 21, 2017 (https://www.drugabuse.gov/about-nida/legislative-activities/testimony-to-congress/2017/research-use-misuse-fentanyl-other-synthetic-opioids), as well as NIDA Director Dr. Volkow's annual report to NIH Council on February 10 (https://www.drugabuse.gov/sites/default/files/ volkowfeb2016council talk.pdf). There has also been extensive coverage in the lay media, including an article on the study by the Associated Press' chief medical writer Marilynn Marchione(https://www.usnews.com/news/news/articles/2016-12-21/a-novel-approach-fighting-painkiller-addiction-at-home). These highly promising pilot data suggest that providing technology-supported IBT to waitlisted opioid-dependent individuals may reduce individual and societal risks during delays to comprehensive treatment, representing substantial potential to produce a large public health impact. Importantly, however, our initial study involved an extremely limited 12-week treatment duration.

Many individuals experience much longer waitlist delays. For example, in our state's first and still largest methadone service, for which Dr. Sigmon is Director, we have increased our capacity from 400 to 1,000 patients in the past several years and yet our clinic still has a waitlist of 6-8 months. Thus, it is essential to evaluate IBT over the durations likely to be encountered in the real world. The proposed RCT will provide critical information on IBT efficacy over the durations of treatment delays that exist in the real world.

References*. Include references to prior human or animal research and references that are relevant to the design and conduct of the study.*

Abrahamsson, T., Widinghoff, C., Lilliebladh, A., Gedeon, C., Nilvall, K., Hakansson, A. (2015). Interim buprenorphine treatment in opiate dependence: A pilot effectiveness study. *Subst Abus.* [Epub ahead of print].

Dunn, K.E., Saulsgiver, K.A., Patrick, M.E., Heil, S.H., Higgins, S.T., & Sigmon, S.C. (2013). Characterizing and improving HIV and hepatitis knowledge among primary prescription opioid abusers. *Drug Alcohol Depend, 133*(2), 625-632.

Fiellin, D.A., Pantalon, M.V., Chawarski, M.C., Moore, B.A., Sullivan, L.E., et al. (2006). Buprenorphine maintenance in primary care: A randomized controlled trial of counseling conditions and medication dispensing. *NEJM, 355*(4), 365-374.

Helzer, J.E., Rose, G.L., Badger, G.J., Searles, J.S.,Thomas, C.S., et al. (2008). Using Interactive Voice Response to Enhance Brief Alcohol Intervention in Primary Care Settings. *J Stud Alcohol Drugs, 69,* 251-258.

Higgins, S.T., Stitzer, M.L., Bigelow, G.E., & Liebson, I.A. (1986). Contingent methadone delivery: Effects on illicit opiate use. *Drug Alc Depend, 17*, 311-322.

Jasinski, D.R., Pevnick, J.S., & Griffith, J.D. (1978). Human pharmacology and abuse potential of the analgesic buprenorphine. *Arch Gen Psychiatry, 35*, 501–516.

Johnson, R.E., Chutaupe, M.A., Strain, E.C., Walsh, S.L., Stitzer, M.L. et al. (2000). A comparison of levomethadyl acetate, buprenorphine, and methadone for opioid dependence. *N Engl J Med, 343*(18):1290-1297.

Johnson, R.E., Strain, E.C., & Amass, L. (2003). Buprenorphine: How to use it right. *Drug Alc Depend, 70*, S59-S77.

Krook, A.L., Brørs, O., Dahlberg, J., Grouff, K., Magnus, P., Røysamb, E., & Waal, H. (2002). A placebo-controlled study of high dose buprenorphine in opiate dependents waiting for medication-assisted rehabilitation in Oslo, Norway. *Addiction, 97*(5), 533-542.

McClure, E.A., Acquavita, S.P., Harding, E., & Stitzer, M.L. (2013). Utilization of communication technology by patients enrolled in substance abuse treatment. *Drug Alc Depend, 129*, 145-150.

Moore, B.A., Fazzino, T., Barry, D.T., Fiellin, D.A., Cutter, C.J., et al. (2013). The Recovery Line: A pilot trial of automated, telephone-based treatment for continued drug use in methadone maintenance. *J Subst Abuse Treat, 45*, 63-69.

Rose, G.L., Skelly, J.M., Badger, G.J., Ferraro, T.A., & Helzer, J.E. (2015b). Efficacy of automated telephone continuing care following outpatient therapy for alcohol dependence. *Addict Behav, 41*, 223-231.

Schwartz, R.P., Alexandre, P.K., Kelly, S.M., O'Grady, K.E., Gryczynski, J., & Jaffe, J.H. (2014). Interim versus standard methadone treatment: A benefit-cost analysis. *J Subst Abuse Treat, 46*(3), 306-314.

Schwartz, R.P., Highfield, D.A., Jaffe, J.H., Brady, J.V., Butler, C.B., et al. (2006). A randomized controlled trial of interim methadone maintenance. *Arch Gen Psychiatry, 63,* 102-109.

Schwartz, R.P., Jaffe, J.H., O'Grady, K.E., Das, B., Highfield, D.A., & Wilson, M.E. (2009a). Scaling-up interim methadone maintenance: Treatment for 1,000 heroin-addicted individuals. *J Subst Abuse Treat, 37,* 362-367.

Schwartz, R.P., Kelly, S.M., O'Grady, K.E., Gandhi, D., & Jaffe, J.H. (2012). Randomized trial of standard methadone treatment compared to initiating methadone without counseling: 12-month findings. *Addiction 107*, 943–952.

Schwartz, R.P., Kelly, S.M., O'Grady, K.E., Gandhi, D., & Jaffe, J.H. (2011). Interim methadone treatment compared to standard methadone treatment: 4-month findings. *J Subst Ab Treat*, 41, 21-29.

Sigmon, S.C. (2015b). Interim treatment: Bridging delays to opioid treatment access. *Prev Med, 80*, 32-36.

Sigmon, S.C., Meyer, A.C., Hruska, B., Ochalek, T., Rose, G., Badger, G.J., Brooklyn, J.R., Heil, S.H., Higgins, S.T., Moore, B.A., & Schwartz, R.P. (2015a). Bridging waitlist delays with Interim Buprenorphine Treatment: Initial feasibility. *Addict Behav, 51*, 136-142.

Sigmon, S.C, Ochalek, T.A, Meyer, A.C, Hruska, B., Heil, S.H, Badger, G.J, … Higgins, S. (2016). Interim buprenorphine vs. waiting list for opioid dependence. *NEJM*, *375*(25), 2504–2505. doi:10.1056/NEJMc1610047

Stacy, J.N., Schwartz, S.M., Ershoff, D., & Shreve, M.S. (2009). Incorporating tailored interactive patient solutions using interactive voice response technology to improve statin adherence: Results of a randomized clinical trial in a managed care setting. *Popul Health Manag, 12*(5), 241-254.

Tacke, U., Uosukainen, H., Kananen, M., Kontra, K., & Pentikäinen, H. (2009). A pilot study about the feasibility and cost-effectiveness of electronic compliance monitoring in substitution treatment with buprenorphine–naloxone combination. *J Opioid Manag, 5*, 321–329.

Uosukainen, H., Pentikainen, H., & Tacke, U. (2013). The effect of an electronic medicine dispenser on diversion of buprenorphine-naloxone: Experience from a medium-sized Finnish city. *J Subst Ab Treat, 45,* 143-147.

Volkow, N.D., Frieden, T.R., Hyde, P.S., Cha, S.S., 2014. Medication-assisted therapies – Tackling the opioid-overdose epidemic. *NEJM, 370*, 2063-2066.

Walsh, S.L., Preston, K.L., Bigelow, G.E., & Stitzer, M.L. (1995). Acute administration of buprenorphine in humans: Partial agonist and blockade effects. *J Pharmacol Exp Ther, 274*, 361-372.

Walsh, S.L., Preston, K.L., Stitzer, M.L., Cone, E.J., & Bigelow, G.E. (1994). Clinical pharmacology of buprenorphine: Ceiling effects at high doses. *Clin Pharmacol Ther, 55,* 569-580.

Objectives: *Clearly state the primary and secondary objective(s) of the study.*

Our primary objective is to determine whether IBT effects in reducing illicit opioid use and other risk behaviors endure long enough to constitute meaningful improvement in people's lives while they await entry into traditional treatment.

METHODS AND PROCEDURES

Study Design: *Describe the research design, including a description of any new methodology and its advantage over existing methodologies.*

We propose to conduct a Stage II randomized trial evaluating the efficacy of IBT using a larger sample of 200 waitlisted opioid-dependent adults and longer duration of evaluation. Participants randomly assigned to the IBT condition (n=100) will receive the above IBT intervention over a 24-week period with monthly assessments. Those randomized to WLC (n=100) will remain on the waitlist for their treatment of choice but complete the same monthly assessments. We hypothesize that IBT participants will demonstrate significantly greater illicit opioid abstinence relative to WLC participants. Additional outcomes will include IV drug use, other (non-opioid) drug use, retention, and several IBT-specific measures (e.g., BUP adherence, IVR utilization, patient satisfaction). WLC participants who have not entered treatment by the end of the study will be offered the opportunity to receive IBT at that time (described below). This study design feature will provide additional data with which to evaluate IBT effects beyond the initial randomized sample of 100 participants.

To begin evaluating IBT generality and scalability, we will partner with federally qualified health centers (FQHCs) in two rural Vermont counties. FQHCs are non-profit health centers that are located throughout the US and have an overarching mission to provide primary care services specifically to medically underserved areas and populations. UVM will serve as the coordinating center and primary recruitment site where we will enroll 100 participants (50 IBT, 50 WLC). Two rural FQHCs will serve as recruitment sites for an additional 100 participants (50 IBT, 50 WLC), with half recruited at each clinic. The clinics are located in Orange and Addison counties, which are similar in terms of population density and median household income (42.1 vs. 48.0 inhabitants per square mile and $52,079 vs. $55,800, respectively). We will establish a mobile IBT team consisting of a nurse and research assistant (RA) who will travel to each clinic to conduct study visits. Considering that IBT involves essentially weekly visits (2 scheduled visits + 2 random call-backs per month), this will be easily accomplished. Our IBT components are also highly transportable (i.e., Med-O-Wheel, IVR monitoring, HIV+Hepatitis iPad application), which will further support the delivery of IBT at sites outside of our centralized university setting. Finally, it is worth noting that, for rural areas that continue to lack sufficient opioid treatment providers, this mobile IBT paradigm could represent an altogether new approach for BUP delivery to individuals living in underserved areas.

Procedures: *Describe all procedures (sequentially) to which human participants will be subjected. Identify all procedures that are considered experimental and/or procedures performed exclusively for research purposes. Describe the types, frequency and duration of tests, study visits, interviews, questionnaires, etc. Include required screening procedures performed before enrollment and while on study. Please provide in table, list or outline format for ease of review. (describe and attach all instruments)*

Note: A clinical research protocol may involve interventions that are strictly experimental or it may involve some aspect of research (e.g., randomization among standard treatments for collection and analysis of routine clinical data for research purposes). It is important for this section to distinguish between interventions that are experimental and/or carried out for research purposes versus those procedures that are considered standard therapy. In addition, routine procedures performed solely for research purposes (e.g., additional diagnostic/follow-up tests) should be identified.

Participants. Participants will be 200 opioid-dependent adults who are waitlisted for agonist maintenance. The primary referral source will be an IRB-approved information sheet, mailed directly to all individuals on the waitlists of the Chittenden Clinic and other local programs, that describes the study and provides a phone number that interested individuals can call to learn more about the study. Additional sources will include the Vermont State Alcohol and Drug Abuse Office, local mental health centers, public service announcements and advertisements in local and alternative newspapers. We have successfully used these methods in prior studies and anticipate no difficulties doing so for this trial (Dunn et al., 2008, 2010; Sigmon et al., 2009, 2013, 2015, under review).

For inclusion, participants must be ≥18 years old, in good health, meet DSM-V criteria for opioid use disorder, provide an opioid-positive urine and be currently waitlisted with a community opioid treatment clinic or provider. Those with a significant psychiatric or medical illness that may interfere with consent or participation will be excluded, as will those who are pregnant or nursing. Females will be tested for pregnancy and, should a participant become pregnant during the trial, her participation will be terminated and she will be assisted with accessing treatment at our hospital's specialty obstetrics clinic. Those dependent on sedative-hypnotics will be excluded, due to the medical risks and notably low success rates with sedative-dependent opioid abusers (Stitzer & Chutuape, 1999). Participants must provide written informed consent to participate. Those meeting the above criteria and interested in IBT will be eligible to participate.

Assessments. Participants will complete an intake assessment that includes a Drug History Questionnaire, ASI (McLellan et al., 1985), psychoactive substance dependence section of the DSM-5 (APA, 2013), Brief Symptom Inventory (Derogatis, 1993), Brief Pain Inventory (Cleeland, 1989, 1990; Keller et al., 2004), Beck Depression and Anxiety Inventories (Beck et al., 1988, 1996), Michigan Alcoholism Screening Test (Selzer, 1971) and a Time-Line Followback of past-month use of opioids and other drugs (Sobell & Sobell, 1992). They will complete a medical history, receive a brief physical exam, and provide a urine specimen for immediate testing (see Biochemical monitoring). We will also obtain participant consent so that we can confirm their waitlist status. We have used these procedures in prior studies (Dunn et al., 2008, 2010; Higgins, Sigmon et al., 2003; Sigmon et al., 2009, 2013, 2015a, under review). Abbreviated versions of the intake, including urinalysis, will be administered monthly. A participant satisfaction questionnaire will also be administered at Week 24. Participants will receive $30 per assessment. This level of compensation has permitted us to achieve high levels of follow-up compliance in our IBT pilot (94%) and prior treatment development (90%) studies.

Interim Buprenorphine Treatment. Participants randomly assigned to the 24-week IBT condition will receive the following treatment components:

Buprenorphine via computerized dispensing. IBT participants will receive buprenorphine/naloxone sublingual tablets (Amneal Pharmaceuticals, Bridgewater, NJ). Consistent with our prior studies, medication will be obtained through our hospital's investigational pharmacy. BUP induction will occur in Week 1, with individualized dose adjustments at daily visits determined using the Clinical Institute Narcotic Assessment (Peachey & Lei, 1988) with the aim of stabilizing participants on a dose sufficient to achieve withdrawal suppression without intoxication or sedation (Sigmon et al., 2009, 2013, 2015, under review). At each visit, an IBT research nurse will assess withdrawal and agonist effects and the RA will collect urine and breath samples. During stabilization, the RA will also instruct IBT participants on how to use the IVR system and Med-O-Wheel and have them complete the HIV+Hepatitis and Overdose Education modules (described below).

Following stabilization, participants will visit the clinic every two weeks (i.e., Weeks 2, 4, 6, etc.) to provide a urine specimen, ingest that day's BUP dose under nurse observation, and complete measures of opioid and other drug use via Time-Line Followback. They will receive the doses for the upcoming interval in a Med-O-Wheel device (Addoz, Forssa, Finland) for ingestion at home. The Med-O-Wheel is a portable pill dispenser with tamper resistant features. Participants will be instructed to bring the device

with them to each study visit, as well as random call-backs (below) and reminded throughout the study that any evidence of nonadherence may be grounds for discharge. Participants can also return to the clinic between scheduled visits should any concerns arise or a dose evaluation be needed. Research staff will work with IBT participants throughout the trial to line up a provider for continued treatment in the community following the study. If a treatment slot is available, we will coordinate a smooth transfer to that program to begin the day following the Week 24 assessment.

IVR daily monitoring. The IVR system will contact participants each evening at a convenient time identified by them to assess opioids and other drug or alcohol use, as well as opioid craving and withdrawal. Instances of use, craving or withdrawal will prompt additional detailed questions (e.g., type and amount used, severity of craving and withdrawal), as well as provision of information about support meetings taking place in the community. Participants can make inbound calls to the system at any time to complete their daily check-in if they anticipate missing the call. They can also reach study staff at any time using our on-call mobile phone.

Random call-backs via IVR. Participants will be contacted via IVR approximately twice per month (once per two-week dosing interval) on a random basis and instructed to return to the clinic within 12 hours. For each random call-back, they will refrain from taking that day's dose and instead bring in their Med-O-Wheel device to ingest their medication under nurse observation. The research nurse will conduct a pill count and inspect the device for any evidence of tampering or nonadherence, and participants will provide breath and urine specimens (see Biochemical monitoring).

HIV+Hepatitis education. Using an interactive iPad application developed by us, during Week 1 participants will complete a baseline assessment of HIV and hepatitis knowledge, including a modified version of the HIV/AIDS Knowledge Test (Marsch et al., 2005), a Hepatitis C knowledge test developed by our group, and VAS items assessing perceived risk, disease transmission and risk behaviors (Dunn et al., 2013). Upon completion of the pre-test, the application will provide immediate corrective feedback of any incorrect items (excluding VAS items) and an explanation of the correct answer. Participants will then view an interactive flipbook (“HIV/AIDS Basics”, Aids.gov) and a brief video (“What is Hepatitis C and how is it diagnosed?”, Foundation for AIDS Research), both delivered by iPad. They will complete a post-test assessment immediately following the educational module, and staff will offer condoms and information on free HIV and hepatitis testing in the community. The knowledge post-test will be repeated at Weeks 12 *and 24*.

Overdose prevention. We will evaluate the OD educational module in a manner similar to the HIV+Hepatitis Education above (i.e., pre-test, intervention, immediate post-test, follow-up post-tests at Weeks 12 and 24). Briefly, during Week 1, participants will complete a baseline assessment of opioid OD knowledge using the BOOK questionnaire (Dunn et al., 2016bc). Participants will then complete a brief (15-min) OD educational application via iPad, which consists of 28 slides that combine text, picture, and/or videos in the above content areas. The knowledge post-test will be repeated immediately following the intervention and at Weeks 12 and 24. We will also assess any instances of OD and naloxone use at each visit.

All participants will receive an emergency naloxone kit containing two doses of naloxone (Narcan®) HCL USP (1mg/mL) in prefilled syringes (Luer-Jet Prefilled Syringe NDC#76329-3369-1), two atomizers for intranasal administration (e.g., Teleflex MAD300/ LMA® MAD Nasal™ Intranasal Mucosal Atomization Device), and a simple instructional brochure. As the above OD education outcome is unrelated to naloxone itself, providing naloxone to participants in both conditions will serve as a safety feature without confounding OD education outcomes. Vermont’s Department of Health is a strong supporter of efforts to distribute emergency naloxone kits to people at risk of OD, as well as family members and others who may be in a position to help in the event of an OD. That office will provide us with the naloxone instructional brochure, sufficient naloxone kits for all participants and in-person training of our staff. The Department of Health staff have extensive expertise with naloxone programming and are happy to provide ongoing consultation on all aspects of the naloxone component.

Biochemical monitoring. Urine specimens will be collected under observation of same-sex staff and immediately analyzed for opioids (methadone, BUP, oxycodone, hydrocodone, hydromorphone, heroin) via handheld CLIA-waived dipsticks (Multi-Drug Screen Test Panel, Innovacon, Inc., San Diego, CA). Samples will also be tested at random intervals for other drugs (e.g., cocaine, amphetamines, benzodiazepines). Biochemical or self-report evidence of recent drug use will not alter study procedures for participants. Should we encounter any problems with the handheld tests, we can also analyze samples using our enzyme multiplied immunoassay machine (Microgenics, Fremont, CA). Finally, participants will

provide a breath sample at each visit to monitor for recent alcohol use (ALCO-SENSOR III, Intoximeters, Inc., St. Louis, MO).

Waitlist Control. Participants randomized to WLC will remain on the waitlist for their treatment of choice but receive the same emergency naloxone kit and monthly assessments as IBT participants. WLC participants will not receive the daily IVR calls and bi-monthly visits described above. Brief comment is warranted on the potential for assessment reactivity to confound group comparisons if there is uneven contact between IBT and WLC groups. One such concern is if the assessments inadvertently function as an intervention, thereby improving the outcomes in the control group and obscuring the effect of the intervention. We saw no evidence in our R34 that completion of monthly assessments inflated biochemically-verified abstinence outcomes in the WLC group (which were 0% at all timepoints), and this is also consistent with a recent study by Co-I Rose showing no reactivity associated with technology-assisted monitoring of alcohol use (Fazzino et al., 2016). Another possibility is that the IVR calls and bi-monthly visits contribute to the IBT outcomes. As these components are part of the IBT intervention rather than assessments of outcome, this would not be a problem. While we think it is unlikely that simply calling participants daily (without BUP) could produce the abstinence rates seen in our pilot study, this is an empirical question that can be evaluated in a future study to dismantle IBT to evaluate the contribution of its individual components. Overall, our aim in the present proposal is to utilize a WLC condition that generally reflects the waitlist conditions received by individuals in the real world, and daily IVR calls and bi-monthly study visits are not a part of the typical waitlist experience.

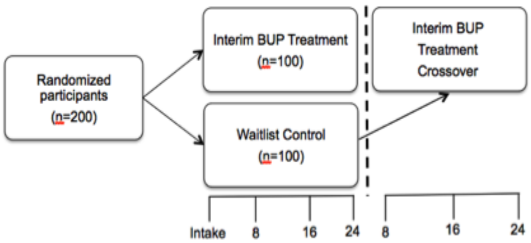

WLC participants who have not entered treatment by the end of the study will be offered the opportunity to receive the 24-week IBT protocol at that time (see Figure). They will receive the same procedures as outlined above for participants originally randomized to IBT (i.e., BUP induction in Week 1, bi-monthly visits and computerized BUP dispensing thereafter). They will receive all other IBT components (e.g., IVR-generated daily monitoring, random call-backs, HIV+Hepatitis and OD education) and complete assessments monthly for 24 weeks.

For research involving survey, questionnaires, etc.: *Describe the setting and the mode of administering the instrument and the provisions for maintaining privacy and confidentiality. Include the duration, intervals of administration, and overall length of participation. (describe and attach all instruments)*

☐ ***Not applicable***

Study procedures will be conducted at UVM Buprenorphine Research Clinic, which has been the site of BUP research for the past 25 years. The clinic is located in our University Medical Center's outpatient building and is contiguous with our other research clinics for cocaine dependence and smoking cessation as well as our methadone clinic for which Dr. Sigmon is Director. IBT participants will complete study visits with research staff every 2 weeks while receiving the IBT package described above. WLC participants will remain on the waitlist for their treatment of choice, though they will complete the same scheduled follow-up assessments as IBT participants. WLC participants who have not entered treatment by Week 24 will be offered the opportunity to cross over to IBT at that time, contributing additional within-subject data with which to evaluate the efficacy of the IBT intervention. The intake assessment will include a Drug History Questionnaire, ASI (McLellan et al., 1985), psychoactive substance dependence section of the DSM-5 (APA, 2013), Brief Symptom Inventory (Derogatis, 1993), Beck Depression and Anxiety Inventories (Beck et al., 1988, 1996), Michigan Alcoholism Screening Test (Selzer, 1971) and a Time-Line Followback of past-month use of opioids and other drugs (Sobell & Sobell, 1992). Participants will also complete a medical history, receive a brief physical exam, and provide a urine specimen. Follow-up assessments will consist of abbreviated versions of the intake, will include urinalysis, and be administered monthly during the 6-month trial (i.e., at Weeks 4, 8, 12, 16, 20, and 24).

Statistical Considerations: *Delineate the precise outcomes to be measured and analyzed. Describe how these results will be measured and statistically analyzed. Delineate methods used to estimate the required number of subjects. Describe power calculations if the study involves comparisons. Perform this analysis on each of the primary and secondary objectives, if possible.*

Statistical methods

IBT and WLC groups will be compared on baseline characteristics using analyses of variance for continuous and chi-square tests for categorical variables. If characteristics differ significantly and are predictive of outcome, they will be considered as covariates in subsequent analyses. The primary outcome

will be the percent of participants in IBT and WLC conditions with biochemically-confirmed illicit opioid abstinence at the 24-week assessment, evaluated based on a Cochran-Mantel-Haenszel (C-M-H) chi square test with site as the stratification variable. Primary analyses will include all randomized subjects independent of early dropout and with missing urine specimens considered positive for illicit opioids, consistent with an intent-to-treat approach (Armitage, 1983). The Breslow-Day test will be used to test homogeneity of the treatment effects across sites. If applicable, logistic regression will be used to adjust for baseline covariates confounded with treatment group. Though not the primary outcome, abstinence at other monthly assessments will be compared across treatments with Bonferroni-adjusted C-M-H tests to control experiment-wise type I error rate below $\alpha=.05$. Within the IBT group, generalized estimating equation (SAS, PROC GENMOD) will be used to characterize the temporal trend in abstinence across assessments. Fixed factors will include recruitment site and the within-subject factor, assessment time, along with their interactions. Repeated measures analyses of variance (SAS, PROC MIXED) will be used to compare groups on continuous outcomes (e.g., past-month frequency of illicit opioid and IV use, ASI subscales, BDI, HIV knowledge, OD risk knowledge). If significant treatment by time interactions are detected, partial F-tests will be used to examine simple effects of treatment at each assessment. Additional repeated measures analyses will characterize temporal patterns in IBT-specific outcomes (e.g., BUP adherence, IVR utilization, satisfaction). For WLC participants who after 24 weeks transition to IBT, estimates of abstinence will be obtained with corresponding 95% confidence intervals at each assessment. Statistical analyses will be performed using SAS V9.4 (SAS Institute, Cary, NC).

Sample size justification

The proposed sample size is based on having sufficient power for detecting treatment differences for primary and secondary outcomes. Regarding the percent of participants negative for illicit opioids at the 24-week assessment, power is estimated to be greater than 95% using $\alpha=.05$ if true abstinence rates are as low as 60% vs. 15% for IBT and WLC groups, respectively. These estimates are more conservative than on our R34 outcomes, with lower abstinence predicted in IBT as we increase the duration to 24 weeks and move beyond a single research clinic for this Stage II trial. For secondary aims, effect sizes range from small to moderate. The estimated differences between conditions in mean change from baseline to 24 weeks corresponding to power=80% for representative ASI subscales are: Employment=.084 (ES=0.23), Psych=.076 (ES=0.36), Legal=.044 (ES=0.32), and Drug=.056 (ES=0.51). Similarly, power is 80% for detecting a mean difference of 4 units in the 24-week change in BDI (ES=0.30) which is smaller than we observed in our pilot trial. Lastly, power is estimated to be 80% to detect a mean absolute difference of 6% in the pre-post change in HIV knowledge scores (ES=0.39) and 5.5% (ES=0.34) for the pre-post change in OD knowledge scores. It should be noted that these ES's (Cohen d's) are expressed relative to the between-subject variability at baseline [i.e. $(\Delta_{IBT}-\Delta_{WLC})/\sigma_{between-subject}$], not the variability in the change scores [i.e. $(\Delta_{IBT}-\Delta_{WLC})/\sigma_{\Delta}$](Becker, 1988; Morris, 2008). The actual computation accounts for the correlation observed across assessments for each outcome observed in our pilot data which is responsible for the different estimates of detectable ES's.

Health economic evaluation

To determine the cost of each condition, we will employ the Brief Drug Abuse Treatment Cost Analysis Program (Brief DATCAP; French et al., 2004), a measure widely used in the area of substance abuse (e.g., French et al., 2008; Roebuck et al., 2003, 2009, 2011; http://datcap.com/). The economic cost of treatment will be derived by allocating fixed costs based upon the proportion of time or space utilized by the programs and costs that vary by patient engagement (e.g., urinalysis, medication). Total cost per treatment episode will be individual-specific and also include the opportunity cost of patient's time as measured by the Client (Outpatient) DATCAP (French, 2005; Salomé et al., 2003). The time period of cost analyses will span from intake to participants' discontinuation or trial completion. Cost of research-specific resources will be excluded. Costs and benefits will be expressed in constant prices. Estimated treatment costs will be combined with the estimated participant healthcare utilization costs to represent total costs per treatment condition. We will conduct benefit-cost (BC) and cost-effectiveness (CE) analyses from the societal perspective (Drummond et al., 2015). Based upon illicit opioid abstinence from Day 1 through the 24-week assessment, the number of quality-adjusted life years (QALYs) gained -as measured by the proportion of time spent in 'abstinent' or 'non-abstinent' status- will be derived (Cromwell et al., 1997; Flack et al., 2007; Zeckhauser & Shepard, 1976). BC and CE ratios will be computed by dividing the mean difference in treatment costs between the two conditions into mean difference in economic benefits or QALYs, respectively. Statistical significance of the ratios will be determined by employing non-parametric bootstrapped standard errors (Drummond et al., 2015). We hypothesize that,

while IBT costs will exceed that of WLC, they will be more than offset by the benefits realized (e.g., reduced health care and criminal justice costs, work days lost, QALYs).

Risks/Benefits: *Describe any potential or known risks. This includes physical, psychological, social, legal or other risks. Estimate the probability that given risk may occur, its severity and potential reversibility. If the study involves a placebo or washout period, the risks related to these must be addressed in both the protocol and consent. Describe the planned procedures for protecting against or minimizing potential risks and assess their likely effectiveness. Where appropriate, discuss plans for ensuring necessary medical or professional intervention in the event of adverse effects to the subjects. Discuss the potential benefits of the research to the subjects and others. Discuss why the risks to the subjects are reasonable in relation to the anticipated benefits to subjects and others. Discuss the importance of the knowledge gained or to be gained as a result of the proposed research and why the risks are reasonable in relation to the knowledge that reasonably may result. If there are no benefits state so.*

Risks include breach of confidentiality and any side effects associated with the study medication (i.e., buprenorphine). Breach of confidentiality: Study data include medical and psychiatric histories and biological measures of alcohol and illicit drug use and pregnancy. The likelihood of a breach of confidentiality is low as we will take precautions to minimize this risk as described below under Adequacy of Protection against Risk.

Side effects of buprenorphine: The side effects of buprenorphine include light-headedness, dizziness, sedation, lethargy, changes in sexual ability, nausea, vomiting, sweating, euphoria, constipation, respiratory depression, flushing of the face, skin itchiness or redness, darkening of the skin and/or swelling, bradycardia, headache, yawning, tearing, runny nose, muscle tremor, dilated or constricted pupil, restlessness, diarrhea, hypertension, hypotension, or potentially elevated liver enzyme levels (particularly among subjects with a history of hepatitis). The administration of the partial opioid agonist, buprenorphine, in individuals physically dependent on opioids should not result in acute toxicity because these individuals are tolerant to such drug effects. There also is a ceiling on the agonist effects of partial agonists; thus, the agonist effects of the partial agonist, buprenorphine, are considered to be safer than full agonists. Because buprenorphine is a partial agonist, it could also function as an antagonist and promote withdrawal symptomatology. We will administer buprenorphine in accordance with standard practice (see Methods) and, based on our previous experience in treating opioid-dependent individuals with this medication (Sigmon et al., 2009; Sigmon et al., 2013, 2015, 2016), we do not anticipate that buprenorphine-precipitated withdrawal or sedation will pose a problem.

Protection Against Risk: (1) To protect confidentiality, the guidelines stated in Title 42 of the Code of Federal Regulations, Part 2, “Confidentiality of Alcohol and Drug Abuse Records” will be followed. As stated in these regulations, subjects will be given a notice of federal confidentiality requirements (which will be included in the consent form). All records will be locked in file cabinets kept on site behind locked doors. Except for intake material, subjects’ names (i.e., first and last names) will not be attached to the data forms. A central code/data base linking subject number with subject names will be kept, which will be available only to specified staff.

(2) In order to protect participants from any adverse effects of buprenorphine, a number of safeguards will be in place. First and most generally, subjects will be screened thoroughly at intake using medical, psychiatric, drug abuse, and cognitive interviews and self-reports. They will have a physical exam and follow-up interviews and tests may be ordered to clarify results. The results of all tests will be reviewed by the medical director (Dr. Brooklyn) and the PI (Dr. Sigmon). Thus, we will document that the patient is healthy to participate in the proposed study. To prevent subjects who may have an insufficient level of opioid dependence from participating in these studies, they will have to meet several criteria (e.g., DSM-5 criteria for opioid dependence and FDA qualification criteria for buprenorphine treatment, including a history of opioid dependence and significant current opioid use). All medication administration will occur during working hours at the University Health Center (UHC), an inpatient/outpatient facility of the University of Vermont College of Medicine and University of Vermont Medical Center. All nursing and research staff will be trained by medical staff in detecting adverse effects. If a subject has any untoward effects, the Study Physician and PI will be contacted. Study Physician Dr. Brooklyn will be on-call continuously for advice and assistance in the event that adverse effects occur. Dr. Brooklyn has been working with our previous buprenorphine projects over the last 15 years, is a buprenorphine provider, is Medical Director of the Chittenden Clinic methadone program, and he has extensive experience with the clinical use of buprenorphine as a Vermont buprenorphine provider and trainer. The emergency room for the University of Vermont Medical Center is located approximately one block away from the UHC.

(3) Finally, patients are free not to participate in this study or to withdraw from it at any time. If they decide not to participate in the study, we will be glad to discuss with them other treatments that may be available in Vermont, including residential, outpatient, and medication-assisted treatment options. If

patients decide not to participate in this study or to withdraw from this study, their decision will not prejudice their future medical care at the University of Vermont or University of Vermont Medical Center. The investigators also retain the right to terminate patients' participation in the study if in their judgment continued participation would put them in physical or psychological danger. Additional details on our data and safety monitoring of the proposed research to ensure the safety of subjects is provided in the below section entitled "Data and Safety Monitoring Plan".

Potential Benefits to Participants and Others

Volunteers may benefit by initiating abstinence from illicit opioids during their study participation, including experiencing a reduction in the wide range of medical, financial, psychosocial, and legal consequences associated with illicit opioid abuse. Volunteers may also benefit from the financial compensation provided as part of the proposed study. By improving treatment access for opioid abuse and dependence, the proposed research also stands to benefit public health in general by reducing the vast economic and societal costs associated with opioid abuse (e.g., health service utilization and costs, criminal activity, contraction of preventable diseases such as HIV and hepatitis). In addition, there are potential scientific benefits to be gained by expanding our empirical knowledge on how to mitigate gaps in treatment access among opioid-dependent individuals. Overall, the individual participant, the medical and scientific communities, and society in general may benefit by our efforts to develop an interim buprenorphine treatment for patients awaiting agonist maintenance. As such, the risks to which individuals may be exposed as a function of their research participation are reasonable in relation to the anticipated benefits.

Importance of the Knowledge to be Gained

The proposed project has the potential to contribute a novel and effective technology-assisted pharmacotherapy protocol that can be widely disseminated to bridge gaps in access to life-saving opioid treatment. Thus, knowledge gained from this research may significantly enhance the accessibility, implementation and effectiveness of drug abuse treatment more generally. Consequently, the risk/benefit ratio is favorable. The risks to which individuals are exposed as a consequence of their research participation are generally less than that associated with continuing their ongoing abuse of illicit opioids. In contrast, the potential and probable benefits to be derived by society in general and by opioid abusers as a group are considerable. In summary, conducting this research seems well justified.

Therapeutic Alternatives: *List the therapeutic alternatives that are reasonably available that may be of benefit to the potential subject and include in the consent form as well.*

☐ ***Not Applicable***

Participants are free not to participate in this study or to withdraw from it at any time. If they decide not to participate in the study, we will be glad to discuss with them other treatments that may be available in Vermont, including residential, outpatient, and medication-assisted treatment options.

Data Safety and Monitoring: *The specific design of a Data and Safety Monitoring Plan (DSMP) for a protocol may vary extensively depending on the potential risks, size, and complexity of the research study. For a minimal risk study, a DSMP could be as simple as a description of the Principal Investigator's plan for monitoring the data and performance of safety reviews or it could be as complex as the initiation of an external, independent Data Safety and Monitoring Board (DSMB). The UVM/UVM Medical Center process for review of adverse events should be included in the DSMP.*

Patient eligibility and status. All intake data collection will be conducted by a trained bachelor's-level Research Assistant (RA) using specialized forms and procedures. Medical screening data will be reviewed by the Study Physician. All intake information will be reviewed by the PI, who will determine participant eligibility. Only trained and IRB-approved research staff will complete informed consent with eligible and willing participants. The status of all active participants will be reviewed at weekly meetings between the PI, Co-investigators and RAs.

Rigorous data management/Quality assurance. The majority of study data collection will be conducted using self-report questionnaires. Randomly selected data will be checked by the RAs for completeness and to ensure quality (i.e., no appearance of rote answers, etc.). In terms of standard operating procedures at the clinic, all assessments will be administered by trained research staff. All subject data will be maintained in secure filing cabinets behind locked doors in order to protect confidential subject information. Safe places will include locked filing cabinets or locked rooms that will be accessible only to study personnel. Full subject names will not be listed on the outside of the binders in order to protect the identity of study participants. Moreover, all data that are entered into spreadsheets and databases, in preparation for data analyses, will be entered twice. That is, two separate individuals will enter the data into databases, and a comparison between data entries will be conducted to detect data entry errors. All discrepancies in data entry will be checked against the raw data source, and the correct data entry will be used. All data that are entered into spreadsheets and databases will be coded by subject

ID number and not by subject name. Additionally, all entered data will be backed up on an external hard drive or a secure project server at least weekly. The biostatistician and PI will discuss any problems at monthly data meetings. Additional meetings will be conducted on an as-needed basis. An original copy of the data will be retained at the clinic should anything happen to the document during transmission.

Auditing procedures. Review of any problems related to quality of data collection, transmission or analyses and of any AEs and SAEs that occurred during the past week will occur at weekly research staff meetings. Interim analyses of efficacy data will be conducted when half the subjects have been entered or at other times based on the joint discretion of the PI, Co-I and biostatistician.

Adverse Event and Unanticipated Problem (UAP) Reporting: *Describe how events and UAPs will be evaluated and reported to the IRB. All protocols should specify that, in the absence of more stringent reporting requirements, the guidelines established in the Committees on Human Research "Adverse Event and Unanticipated Problems Reporting Policy" will be followed. The UVM/UVM Medical Center process for review of adverse events and UAPs to subjects or others should be included in the DSMP.*

AEs and SAEs will be assessed at each clinic visit by a trained RA or nurse. Any SAE will be brought to the attention of the PI as soon as possible and not longer than 24 hrs. Any SAE, whether or not related to study intervention, will be reported to the IRB using the UVM Adverse Event Reporting Document within 5 days of the event. The IRB will make a determination as to whether additional reporting requirements are needed. Any SAEs will also be summarized in the yearly IRB continuing review, including a review of frequency and severity.

Withdrawal Procedures: *Define the precise criteria for withdrawing subjects from the study. Include a description of study requirements for when a subject withdraws him or herself from the study (if applicable).*

Completing study visits and providing urine specimens as scheduled are necessary in order for subjects to receive the study medication. Thus, if the subject fails to attend scheduled visits, or does not submit regularly scheduled urine specimens, or if there is any evidence of nonadherence to the medication regimen, they will be withdrawn from the study. If they are discharged from the study, subjects will be expected to return all remaining medication and the Med-O-Wheel device to study staff immediately. Females will be tested for pregnancy prior to and during the study. Should a subject become pregnant during the trial, her participation will be terminated and she will be assisted with accessing treatment at the medical center's high-risk pregnancy clinic. Patients are free not to participate in this study or to withdraw from it at any time. If they decide not to participate in the study, we will be glad to discuss with them other treatments that may be available in Vermont, including residential, outpatient, and medication-assisted treatment options. If patients decide not to participate or to withdraw from this study, their decision will not prejudice their future medical care at UVM or UVM Medical Center.

Sources of Materials: *Identify sources of research material obtained from individually identifiable human subjects in the form of specimens, records or data. Indicate whether the material or data will be obtained specifically for research purposes or whether use will be made of existing specimens, records or data.*

Research materials will include questionnaires, structured clinical interviews, expired air samples for analyzing breath alcohol levels, urine samples for analyzing recent drug use and pregnancy status. All data will be collected for research purposes only. All data collection will be conducted by a trained bachelor's-level Research Assistant (RA) with special training on all forms and procedures. All information will be reviewed by the PI, who will determine participant eligibility and complete informed consent with eligible and willing participants. Subject data will be maintained in secure filing cabinets behind locked doors in order to protect confidential subject information. Safe places will include locked filing cabinets or locked rooms that will be accessible only to study personnel. Full subject names will not be listed on the outside of the binders in order to protect the identity of study participants. Subject data and subject identifiers will only be accessible to approved research staff.

DRUG AND DEVICE INFORMATION

Investigators are encouraged to consult the UVM Medical Center Investigational Pharmacy Drug Service (847-4863) prior to finalizing study drug/substance procedures.

Drug (s) ☐ ***Not applicable***

Drug name – generic followed by brand name and common abbreviations. Availability – Source and pharmacology; vial or product sizes and supplier. If a placebo will be used, identify its contents and source. (attach investigational drug brochure)

IBT participants will receive buprenorphine/naloxone sublingual tablets (Amneal Pharmaceuticals, Bridgewater, NJ), which will be obtained through the UVM Medical Center's Investigational Pharmacy. Buprenorphine is an FDA approved and widely-used medication for treating opioid use disorder.

Preparation: Reconstitution instructions; preparation of a sterile product, compounded dosage form; mixing guidelines, including fluid and volume required. Identify who will prepare.

UVM Medical Center Outpatient Pharmacy at University Health Center

Storage and stability – for both intact and mixed products.

Administration – Describe acceptable routes and methods of administration and any associated risks of administration.

Daily sublingual administration

Toxicity – Accurate but concise listings of major toxicities. Rare toxicities, which may be severe, should be included by indicated incidence. Also adverse interactions with other drugs used in the protocol regimen as well as specific foods should be noted. Address significant drug or drug/food interactions in the consent form as well. List all with above details.

No major toxicities

Is it FDA approved: (include FDA IND Number)

1. *in the dosage form specified? If no, provide justification for proposed use and source of the study drug in that form.*
   Y
2. *for the route of administration specified? If no, provide justification for route and describe the method to accomplish.*
   Y
3. *for the intended action?*
   Y

Device (s) [x] ***Not applicable***

Device name and indications (attach investigational device brochure)

Is it FDA approved: (include FDA IDE Number)

1. *for indication specified? If no, provide justification for proposed use and source of the device.*

Risk assessment (non-significant/significant risk) - PI or sponsor needs to assess risk of a device based upon the use of the device with human subjects in a research environment.

SUBJECT CHARACTERISTICS, IDENTIFICATION AND RECRUITMENT

Subject Selection: *Provide rationale for subject selection in terms of the scientific objectives and proposed study design.*

Subjects will be adults with current opioid use disorder who are waitlisted for methadone or buprenorphine treatment

Vulnerable Populations: *Explain the rationale for involvement of special classes of subjects, if any. Discuss what procedures or practices will be used in the protocol to minimize their susceptibility to undue influences and unnecessary risk (physical, psychological, etc.).*

[x] ***Not applicable***

Number of Subjects: *What is the anticipated number of subjects to be enrolled at UVM/UVM Medical Center and in the case of a multi-center study, with UVM/UVM Medical Center as the lead, the total number of subjects for the entire study.*

The anticipated number of subjects to be enrolled in the study is 200 (100 per experimental condition).

Inclusion/Exclusion Criteria: *Eligibility and ineligibility criteria should be specific. Describe how eligibility will be determined and by whom. Changes to the eligibility criteria at a later phase of the research have the potential to invalidate the research.*

Participants must be ≥18 years old, in good health, meet DSM-5 criteria for opioid use disorder and provide an opioid-positive urine at study intake. Consistent with our prior BUP studies, those with a significant psychiatric or medical illness that may interfere with consent or participation will be excluded, as will those who are pregnant or nursing. Those dependent on sedative-hypnotics will be excluded, due to the medical risks and notably low success rates with sedative-dependent opioid abusers (Stitzer & Chutuape, 1999). Intake information will be reviewed by the PI (Dr. Sigmon) and study physician (Dr. Brooklyn), who will determine and document whether the participant is eligible and healthy to participate.

Inclusion of Minorities and Women: *Describe efforts to include minorities and women. If either minorities or women are excluded, include a justification for the exclusion.*

The study will include minorities and women. We will do all that we can to assure that women and minorities are represented in the research, including using a wide range of recruitment sources to ensure that information regarding this project reaches as diverse of a population as possible (e.g., flyers posted in multiple local neighborhoods, advertisements in the widely-distributed primary local newspaper as well as alternative local papers).

Inclusion of Children: *Describe efforts to include children. Inclusion is required unless a clear and compelling rationale shows that inclusion is inappropriate with respect to the health of the subjects or that inclusion is inappropriate for the purpose of the study. If children are included, the description of the plan should include a rationale for selecting or excluding a specific age range of children. When included, the plan must also describe the expertise of the investigative team in working with children, the appropriateness of the available facilities to accommodate children, and the inclusion of a sufficient number of children to contribute to a meaningful analysis relative to the purpose of the study.* ***If children are excluded*** *then provide appropriate justification. Provide target accrual for this population.*

Children will not be eligible for this study.

For protocols including the use of an investigational drug, indicate whether women of childbearing potential have been included and, if not, include appropriate justification.

N/A

If HIV testing is included specifically for research purposes explain how the test results will be protected against unauthorized disclosure. Include if the subjects are to be informed of the test results. If yes, include the process and provision for counseling. If no, a rationale for not informing the subjects should be included.

x	***Not applicable***

Recruitment: *Describe plans for identifying and recruitment of subjects. All recruitment materials (flyers, ads, letters, etc) need to be IRB approved prior to use.*

The primary referral source will be distribution of an IRB-approved flyer to all CC waitlist individuals informing them about the study. Should this recruitment method ever become insufficient, we can also circulate ads throughout the larger community in order to reach additional patients on wait lists for BUP maintenance via OBOT. Additional sources will include self-referrals, drug abuse clinics, the Vermont State Alcohol and Drug Abuse Office, physicians, local mental health centers, a toll-free number, public service announcements, advertisements in local and alternative newspapers and flyers placed throughout the community. We have successfully recruited participants using these sources in prior studies (Dunn et al., 2008, 2010; Sigmon et al., 2009, 2013, 2015, 2016) and anticipate no difficulties gaining ready access to the sample needed.

Contact between participants and study staff will be initiated by the participants. Potential participants will respond to mailings or advertisements that contain a study description and the name and phone number of the Research Assistant. When potential participants call the Research Assistant, s/he will briefly describe the study and use a brief phone screen to make a preliminary determination about the potential participant’s eligibility. Those who are interested in participating and appear to be eligible will be schedule for a longer intake screening that will begin with a full study description of study procedures. Those interested in undergoing study screening will then be provided with a copy of the consent form to read as we go over it with them. Risks and benefits of the study will be described. Potential participants will be asked to paraphrase the consent form and will be asked questions to determine their understanding of key elements of the informed consent. Potential participants who wish to proceed with the interview will be asked to sign the interview consent form and will be given a signed copy of his/her signed consent form.

FINANCIAL CONSIDERATIONS

Expense to Subject: *If the investigation involves the possibility of added expense to the subject (longer hospitalization, extra studies, etc.) indicate in detail how this will be handled. In cases where the FDA has authorized the drug or device company to charge the patient for the experimental drug or device,* ***a copy of the authorization letter from the FDA or sponsor must accompany the application. Final approval will not be granted until the IRB receives this documentation.***
There are very limited circumstances under which study participants may be responsible (either directly or via their insurance) for covering some study-related expenses. If the study participant or their insurer(s) will be billed for any portion of the research study, provide a justification as to why this is appropriate and acceptable. For example, if the study involves treatment that is documented standard of care and not investigational, state so. In these cases, the protocol and the consent should clearly define what is standard of care and what is research.

There are no expenses to the subject for any aspect of the study.

Payment for participation: *Describe all plans to pay subjects, either in cash, a gift or gift certificate. Please note that all payments must be prorated throughout the life of the study. The IRB will not approve a study where there is only a lump sum payment at the end of the study because this can be considered coercive. The amount of payment must be justified. Clarify if subjects will be reimbursed for travel or other expenses.*

	Not applicable

Participants will receive a $30 gift certificate for completing each assessment (at intake and 4, 8, 12, 16, 20, and 24-week follow-up assessments).

Collaborating Sites*. When research involving human subjects will take place at collaborating sites or other performance sites when UVM/UVM Medical Center is the lead site, the principal investigator must provide in this section a list of the collaborating sites and their Federalwide Assurance numbers when applicable. (agreements may be necessary)*

x	***Not applicable***

INFORMED CONSENT

***Consent Procedures**: Describe the consent procedures to be followed, including the circumstances under which consent will be obtained, who will seek it, and the methods of documenting consent. Specify the form(s) that will be used e.g. consent (if multiple forms explain and place identifier on each form), assent form and/or HIPAA authorization (if PHI is included). These form(s) must accompany the protocol as an appendix or attachment.*
Note: Only those individuals authorized to solicit consent may sign the consent form confirming that the prospective subject was provided the necessary information and that any questions asked were answered.

Only the site-PI (Dr. Sigmon) or trained, IRB-approved research staff will obtain informed consents for these studies. Informed consent will be initiated in a private office in the UHC building. Participants will be given unlimited time to decide if they wish to participate. The site-PI (Dr. Sigmon) and/or trained, IRB-approved research staff will be present and the PI will be continuously available to answer questions and explain all study procedures at any time.

***Information Withheld From Subjects:** Will any information about the research purpose and design be withheld from potential or participating subjects? If so, explain and justify the non-disclosure and describe plans for post-study debriefing.*

x	***Not applicable***

Summary of important changes after trial commencement (Trial 2):

1.) Given changes in treatment availability in Vermont between the pilot and present studies, the inclusion criterion that participants be waitlisted for agonist maintenance was modified to include participants not currently receiving opioid agonist treatment.

2.) Following an initial request (prior to trial commencement) by the funding agency to increase the originally-proposed 12-week duration to a similar 24-week duration in order to parallel that of Trial 1, the proposed sample size was then also reduced to align with that of parallel Trial 1.

3.) HIV, HCV, and opioid overdose knowledge post-tests were added to the Week 4 assessment.

4.) Compensation for intake and monthly assessments was increased from $30 to $75 to facilitate timely recruitment.

5.) Remote study sites were broadened to include locations that were convenient to participants (e.g., municipal office, fire station, community substance treatment facility).

6.) With the onset of the COVID-19 pandemic, the following modifications were made to the study protocol in situations where participants were at high-risk and/or currently experiencing COVID-19 symptoms: participants were permitted meet with study staff less frequently or remotely via phone or UVM's institutionally-supported Zoom platform to reduce the risk of COVID-19 transmission; biochemical samples may be omitted or collected using modified procedures to reduce risk of COVID transmission.
