# Supplementary material for: Technology-Assisted Buprenorphine Treatment in Rural and Nonrural Settings: Two Randomized Clinical Trials
Source: JAMA Netw Open. 2023 Sep 27;6(9):e2331910. doi: 10.1001/jamanetworkopen.2023.31910 (PMC10534272; doi:10.1001/jamanetworkopen.2023.31910)
Supplement: Supplement 2. — Data Sharing Statement [file jamanetwopen-e2331910-s002.pdf]

Data Sharing Statement

Sigmon. Technology-Assisted Buprenorphine Treatment in Nonrural and Rural Settings. *JAMA Netw Open*. Published September 07, 2023. doi:10.1001/jamanetworkopen.2023.31910

Data
Data available: Yes
Data types: Deidentified participant data
How to access data: All data requests should be submitted to the corresponding author (Stacey.Sigmon@uvm.edu) for consideration.
When available: beginning date: 05-01-2025

Supporting Documents
Document types: None

Additional Information
Who can access the data: The data will be made available to researchers whose proposed use of the data has been approved.
Types of analyses: The data will be made available for research purposes.
Mechanisms of data availability: The data will be made available without investigator support after approval of a proposal.
